# Identifying the role of PrimPol in TDF-induced toxicity and implications of its loss of function mutation in an HIV+ patient

**DOI:** 10.1038/s41598-020-66153-z

**Published:** 2020-06-09

**Authors:** Vincent N. Duong, Lei Zhou, María I. Martínez-Jiménez, Linh He, Moises Cosme, Luis Blanco, Elijah Paintsil, Karen S. Anderson

**Affiliations:** 1 Department of Pharmacology, Yale School of Medicine, 06510 New Haven, Connecticut USA; 2 Department of Pediatrics, Yale School of Medicine, 06510 New Haven, Connecticut USA; 3 Centro de Biología Molecular Severo Ochoa, CSIC-UAM, 28049 Madrid, Spain; 4 Department of Epidemiology & Public Health, Yale School of Medicine, 06510 New Haven, Connecticut USA; 5 Department of Molecular Biophysics and Biochemistry, Yale University, 06510 New Haven, Connecticut United States of America

**Keywords:** Mitochondrial proteins, Enzymes, Drug safety, Translational research

## Abstract

A key component of antiretroviral therapy (ART) for HIV patients is the nucleoside reverse transcriptase inhibitor (NRTI) is tenofovir. Recent reports of tenofovir toxicity in patients taking ART for HIV cannot be explained solely on the basis of off-target inhibition of mitochondrial DNA polymerase gamma (Polγ). PrimPol was discovered as a primase-polymerase localized to the mitochondria with repriming and translesion synthesis capabilities and, therefore, a potential contributor to mitochondrial toxicity. We established a possible role of PrimPol in tenofovir-induced toxicity *in vitro* and show that tenofovir-diphosphate incorporation by PrimPol is dependent on the n-1 nucleotide. We identified and characterized a PrimPol mutation, D114N, in an HIV+ patient on tenofovir-based ART with mitochondrial toxicity. This mutant form of PrimPol, targeting a catalytic metal ligand, was unable to synthesize primers, likely due to protein instability and weakened DNA binding. We performed cellular respiration and toxicity assays using PrimPol overexpression and shRNA knockdown strains in renal proximal tubular epithelial cells. The PrimPol-knockdown strain was hypersensitive to tenofovir treatment, indicating that PrimPol protects against tenofovir-induced mitochondrial toxicity. We show that a major cellular role of PrimPol is protecting against toxicity caused by ART and individuals with inactivating mutations may be predisposed to these effects.

## Introduction

Human immunodeficiency virus (HIV) treatment and chemoprophylaxis regimens commonly consist of one or more nucleoside reverse transcriptase inhibitors (NRTIs) that target the HIV-1 reverse transcriptase enzyme. NRTIs are nucleoside analogs that lack a 3′-OH, thus acting as chain terminators of viral replication once incorporated by HIV reverse transcriptase (RT). Since the approval of zidovudine (AZT) in 1987, NRTIs have become the backbone of antiretroviral therapy (ART); the advent of ART has led to sustained HIV viral suppression, dramatic decrease in HIV-associated morbidity, and mortality^1,2^. Thus, contemporary NRTI-based regimens have significantly contributed to the health of HIV+ individuals allowing them to have near-normal or normal life expectancies as the general population in the absence of a cure for HIV^2–5^.

NRTI toxicity has widely been attributed to off-target inhibition of the primary polymerase responsible for mitochondrial genome replication, DNA Polymerase gamma (Polγ), sometimes termed the Polγ hypothesis^6–8^. Particularly with earlier generation NRTIs, these off-target effects could lead to lactic acidosis, lipodystrophy, peripheral neuropathies, cardiomyopathies, skeletal muscle myopathies, and pancytopenia^9–11^. However, there are discrepancies between the degree of *in vitro* inhibition of Polγ and the observed clinical toxicity of certain NRTIs. For instance, the NRTI tenofovir disoproxil fumarate (TDF), a prodrug of tenofovir, is among the least toxic inhibitors of Polγ as determined by *in vitro* assays, however, there are reports of mitochondrial dysfunction and toxicity by TDF in the renal proximal tubules of the kidneys of HIV-infected individuals^12–14^. Mechanistic studies have shown that Polγ incorporates the natural dATP substrate much more efficiently and selects against the active tenofovir diphosphate (TFV-DP) metabolite leading to a very favorable *in vitro* discrimination factor, suggesting that the Polγ hypothesis cannot fully explain the proposed mitochondrial toxicity caused by TDF^15,16^. These discrepancies may be explained by factors such as differences in metabolism, binding affinity and rate of incorporation of the respective NRTIs by Polγ, ineffective exonuclease removal, and the role of additional host cell polymerases^17–19^.

PrimPol is the most recent enzyme involved in DNA replication that has been observed to be localized to the mitochondria apart from Polγ^20–24^. Characterization of PrimPol has revealed that it is a DNA and RNA primase as well as a DNA-dependent translesion synthesis polymerase^20,21^. Further evidence has implicated that the primary role of PrimPol *in vivo* is repriming stalled replication forks by hydroxyurea (HU) or UV light^23,25^, rising from G-quadruplexes^26^, R-loops^27^, or chain-terminating nucleotides^25–27^. We have previously confirmed that PrimPol is able to incorporate a subset of NRTIs^28^, establishing a potential role of PrimPol in NRTI-induced mitochondrial toxicity. The possible involvement in toxicity could be magnified by mutations in PrimPol or Polγ that impair catalytic function. In fact, prior studies from our lab identified a Polγ R953C mutant in an HIV+ patient, which may predispose the patient to NRTI-induced mitochondrial toxicity by altering the ability of Polγ to discriminate between natural nucleotides and NRTI nucleotides^29^. We postulated that if variants of PrimPol that impair the function of PrimPol existed in individuals, then these mutations could predispose these individuals to possible NRTI-induced toxicity.

Based upon the earlier finding that a mutation in Polγ may predispose patients on NRTI-regimens, we sought to identify possible mutations in the *PRIMPOL* gene in a cohort of HIV+ patients experiencing mitochondrial toxicity under tenofovir-containing antiretroviral drug regimens. We identified an HIV+ patient in this cohort who had a D114N mutation in PrimPol. In the current study, we characterized the effects of D114N PrimPol mutation at the molecular level and found that this amino acid substitution substantially impairs the primase and polymerase catalytic activities. Taking into consideration the repriming capabilities of PrimPol and the potential for off-target incorporation of NRTIs by host polymerases, we began by addressing the broader question of whether PrimPol may directly contribute to NRTI-induced mitochondrial toxicity with a focus on TDF. We validated that PrimPol was able to incorporate the active form of tenofovir (TVF-DP) *in vitro*, albeit with a relatively low efficiency. Then we generated PrimPol overexpression and knockdown renal proximal tubular epithelial cells (RPTECs) to assess mitochondrial toxicity and respiration when the cells were treated with TDF. Under our experimental conditions, we propose that PrimPol plays a protective role against NRTI-induced toxicity. We surmise that the presence of inactivating mutations in PrimPol such as D114N might contribute to the mitochondrial toxicity associated renal toxicity in some patients on TDF-based ART.

## Results

### PrimPol has low efficiency of tenofovir diphosphate incorporation

The current study is focused on defining potential mechanisms of NRTI-mediated nephrotoxicity in HIV+ patients who are taking tenofovir-containing antiretroviral drug regimens. Since TDF has been shown to only be a weak inhibitor of mitochondrial Polγ^15^, we examined a potential role of PrimPol, a primase-polymerase, found to have significant levels of expression in the kidney^30^.

**Figure 1:**
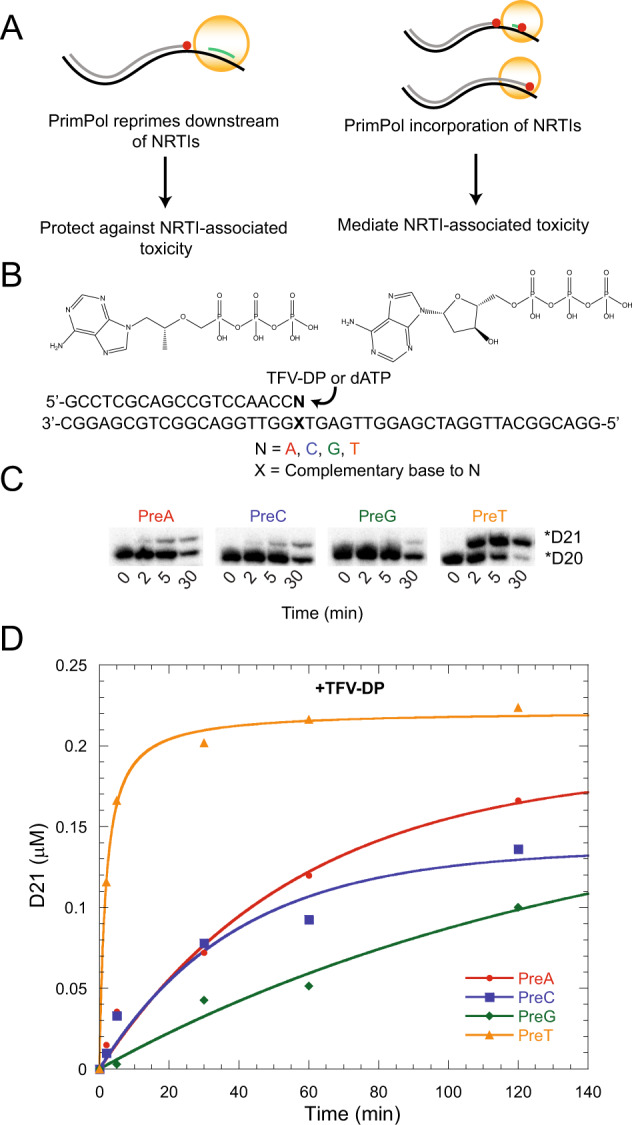
PrimPol modestly incorporates tenofovir-diphosphate *in vitro* with a preceding nucleotide preference. (**A**) Diagram depicting the potential roles of PrimPol in NRTI-associated toxicity. The left panel demonstrates the ability of PrimPol to alleviate toxicity by repriming downstream of a chain-terminated strand. In the right panel, PrimPol can mediate toxicity by incorporating NRTIs and thus stalling replication. Alternatively, the incorporation of NRTIs could prevent the ability of PrimPol to rescue replication by terminating priming. (**B**) Experimental reaction set-up to demonstrate tenofovir-diphosphate incorporation by PrimPol. Generally, a radiolabeled dsDNA substrate with a template dT in the next incorporation position is extended by either dATP or TFV-DP. The n-1 nucleotide and its complimentary base was varied (referred to as PreA, PreC, PreG, PreT) to show the effect on efficiency of nucleotide incorporation. (**C**) Denaturing PAGE of the TFV-DP incorporation reaction with varying nucleotides in the position preceding incorporation. The lower band is the initial substrate and the upper band is the TFV-DP-incorporated DNA. (**D**) Graphical representation of the reaction shown in C). See also Fig. S1.

A role of PrimPol is to reprime downstream of stalled replication forks, which may arise due to depletion in dNTPs, thymine-dimers formation by UV, G-quadruplexes, R-loops, or chain-terminating nucleotides, during both nuclear and mitochondrial DNA replication^23,25–27,31^. Thus, in the context of the mitochondrial toxicity associated with NRTI-based therapies, PrimPol can have a protecting role by repriming and rescuing replication forks that were stalled due to NRTI incorporation by Polγ (Fig. 1A, left). Alternatively, PrimPol could directly contribute to NRTIs-associated toxicity, taking into consideration these nucleotide analogues could also be valid substrates for PrimPol that could conceivably block its primase/polymerase activity (Fig. 1A, right). In this event, PrimPol could increase toxicity via chain termination, by the synthesis of abortive primers and the consequent inability to rescue stalled forks. Our previous work has confirmed the incorporation of select NRTIs by PrimPol as ddATP or CBV-TP, the active metabolites of didanosine (ddI) and abacavir (ABC), with discrimination values in the efficiency of the incorporation (efficiency_dNTP_/efficiency_NRTI_) from 3 to 10^2^-fold^28^. In the current study, we investigated the likelihood that tenofovir could be utilized as a substrate by PrimPol during elongation, taking into consideration that there is a rising, unexplained renal toxicity in patients taking TDF-based ART.

We tested the incorporation of the active form of TDF, tenofovir-diphosphate (TFV-DP), compared to the natural nucleotide dATP, using a defined labeled primer/template. In light of recent findings with other polymerases, indicating that the nucleotide directly adjacent to the incorporation site (n-1) may plausibly affect substrate binding^32^, we used 4 variants of the primer/template, differing in the base pair (N:X) forming the primer-terminus (Fig. 1B). We currently term these substrates as PreA, PreC, PreG, and PreT, corresponding to a dA, dC, dG, and dT in the n-1 position of the primer. In conducting these biochemical assays, we observed that PrimPol can incorporate TFV-DP differently on these substrates, showing more efficient kinetics of incorporation in favor of PreT (Figs. 1C,D, and S1A). We observed the appearance of lower length DNA bands in our polyacrylamide gel at longer timepoints in the incorporation reactions, suggesting that the protein purification resulted in a minor exonuclease contamination (Fig. S8). However, because identical amounts of protein were used in each reaction, the densities of the bands corresponding to the amount of product formed is still indicative of the preferred primer for TFV-DP incorporation. We validated these results with a different pair of primer and template, demonstrating that this effect is primarily due to the n-1 nucleotide (Figs. S1B and S9A). Furthermore, we also observed that this effect persists when the zinc finger domain of PrimPol is absent, which suggests that active site interactions in the polymerase domain may be able to explain the preceding nucleotide preference (Figs. S1C, S9B). Testing other active triphosphate NRTIs that PrimPol incorporates, d4T, (−)-3TC, and (−)-FTC, shows that this effect is unique to tenofovir (Figs. S1D, S9C–E).

As a control to determine if this preceding nucleotide preference is unique to tenofovir or also shared with the natural nucleoside substrate, we generated full *K*_d_ curves through single-turnover kinetics for TFV-DP and dATP incorporation by PrimPol with all four preceding nucleotide variations in the template (Table 1, Fig. S8A–F). The efficiency of TFV-DP incorporation as a function of the preceding nucleotide was PreT> PreA> PreG ~ PreC, with a 10-fold variation between PreT and PreC. However, considering this preference effect with natural dATP incorporation shows a difference of only about 2-fold. This observation implies that the insertion of TFV-DP is facilitated somehow by a thymine base at the primer-terminus. However, even in this favored context the insertion of the TFV-DP is 2 × 10^4^-fold lower than the insertion of the dATP counterpart. In comparison to previously determined incorporation efficiencies of other NRTIs, TFV-DP incorporation is weaker, being incorporated approximately three magnitudes less efficiently than CBV-TP (Table S1)^28^.

**Table 1:** Summary of TFV-DP and dATP incorporation by WT PrimPol dependent on the preceding nucleotide in the primer strand.

TFV-DP	k_pol_ (s^−1^)	K_d_ (µM)	Inc. Eff. k_pol_/K_d_ (s^−1^ µM^−1^)	Fold-difference to PreC
PreA	0.0016 ± 0.0003	85.6 ± 8.3	1.8 × 10^−5^	2.4
PreC	0.0013 ± 0.0002	172.3 ± 15.3	7.6 × 10^−6^	1
PreG	0.0014 ± 0.0005	138.1 ± 43.7	1.0 × 10^−5^	1.3
PreT	0.0037 ± 0.001	45.0 ± 13.7	8.2 × 10^−5^	10.8
dATP				
PreA	1.3 ± 0.06	0.7 ± 0.3	1.9	2.7
PreC	3.2 ± 0.2	4.6 ± 1.3	0.7	1
PreG	0.6 ± 0.03	0.7 ± 0.2	0.9	1.3
PreT	4.6 ± 0.3	2.9 ± 0.9	1.6	2.3

Pre-steady state kinetic parameters for both TFV-dP and dATP incorporation by WT PrimPol at at 37 °C were determined by fitting the time course data to the following single exponential equation: [*product*] = *A*(1− *e*^−*k*^ *obs*^*t*^), where A is amplitude and k_*obs*_ is the observed single exponential rate, and t is the time. In the case of dATP incorporation, the single exponential rates were then plotted against each concentration of [dNTP] using a quadratic equation in order to extract the k_pol_, the maximal rate of incorporation, K_d_, the apparent binding constant for the incoming nucleotide, and k_pol_/K_d_, the overall efficiency for nucleotide incorporation. In the case of TFV-DP, the k_pol_ was independent of [dNTP] the amplitude was plotted against [dNTP] and fit to the quadratic equation to determine the K_d_ value. The errors represent the standard error values of the parameters that corresponds to a confidence level of 68.3%, or to one standard deviation. See also Fig. S1.

Lastly, we addressed the possibility that TFV-DP could hinder the priming activity of PrimPol. We assessed the ability of TFV-DP to compete with ATP during dimer formation on a 3′-T_20_G**TC**AGACAGCAT_29_-5′ substrate. Even at concentrations of TFV-DP in excess of ATP, dimer formation was not disrupted (Fig. S3A). Next, we examined the ability of TFV-DP to interrupt primer elongation using various templates and substrates (Fig. S3B). Extremely high concentrations of TFV-DP were able to reduce the primer length of the products to a greater extent when competing against ATP compared to dATP. This is likely because PrimPol prefers to utilize dATP when elongating primers. We also observe that TFV-DP is able to modestly reduce the length of products when dATP is in either the 3′ site, or 5′ site. Together, the weak interference of priming activity and modest efficiency of NRTI incorporation suggests that PrimPol would likely be a lesser contributor to TDF toxicity.

### Identifying the D114N PrimPol mutation in an HIV+ patient with mitochondrial toxicity

Our previous studies identified a Polγ mutation in an HIV+ patient that appeared to confer an increased susceptibility to mitochondrial-associated toxicity due to NRTI-based antiretroviral therapy, in particular, 3TC (lamivudine)^29^. We sought to identify possible mutations in the *PRIMPOL* gene in a cohort of HIV+ patients with ART-induced mitochondrial toxicity. The current PrimPol study is a subanalysis of mitochondrial toxicity study that enrolled participants at the Yale-New Haven Hospital from April 2011 to March 2013. The details of the study design for this cohort have been described previously^33^. In brief, for this PrimPol sub-study, cases (n = 13) comprised HIV-infected individuals on ART for at least 12 months with clinical and/or laboratory toxicities associated with mitochondrial toxicity. Cases were matched by age, sex, and race/ethnicity to HIV-negative controls (n = 19). All participants gave their written informed consent before participation in the study. The study protocol was approved by the Institutional Review Board of the Yale School of Medicine and all the research was performed in accordance to the relevant guidelines and regulations.

**Figure 2:**
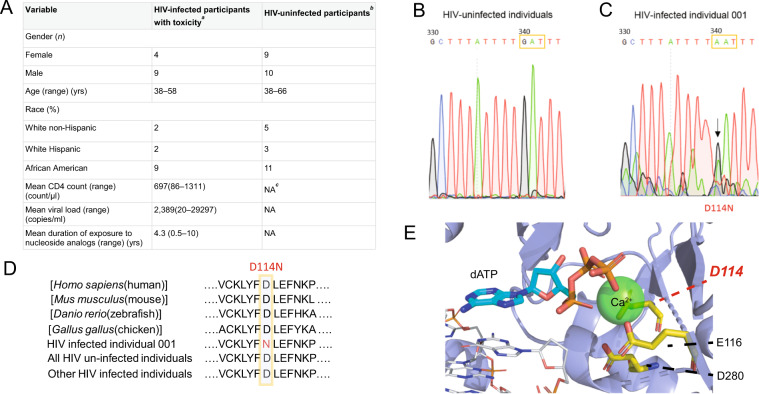
Identification of the D114N active site mutation in an HIV-positive patient experiencing nephrotoxicity. (**A**) Patient data table of a cohort of 13 HIV-infected patients experiencing toxicity under a tenofovir-containing therapy compared to 19 HIV-uninfected patients. *a*. n = 13, *b*. n = 19, *c*. NA, not applicable. (**B**) Sanger sequencing results of the PrimPol gene in a healthy control and a (**C**) HIV-infected individual 001 showing a heterozygous mutation of g.340->a.340 resulting in a D114N mutation in the protein. (**D**) Protein sequence alignment of HIV patient 001 compared to the PrimPol gene of various species and to other patients in the cohort study. (**E**) Active site of the PrimPol crystal structure demonstrating the role of D114 as a catalytic residue (yellow) that coordinates a divalent metal ion (green). PDB: 5L2X.

The demographic and HIV disease characteristics of study participants are illustrated in Fig. 2A. Archived peripheral blood mononuclear cells (PBMCs) of the participants were used for sequencing of *PRIMPOL (*also named as *Ccdc111)*. At enrollment CD4 counts, viral load, and duration of exposure to NRTI-containing therapies were extracted from their medical records. Upon Sanger sequencing of the conserved active site and zinc finger domain regions of *PRIMPOL* of the cohort, we observed heterogeneity within the genomic sequence of a patient compared to healthy individuals (Fig. 2B,C). We concluded that this patient, referred to as individual 001, possesses a heterozygous g.340 transition mutation to a.340, translating into a D114N mutation at the protein level. Protein sequence alignment of PrimPol across different species and other individuals in the cohort emphasize the conservation of D114 (Fig. 2D). Given the importance of D114 as a key residue in the catalytic triad of PrimPol that coordinates a divalent metal ion (Fig. 2E)^34,35^, we hypothesized that D114N is a hindering mutation that impairs the overall activity of PrimPol in individual 001.

### The PrimPol D114N mutation is deficient in primase activity

**Figure 3:**
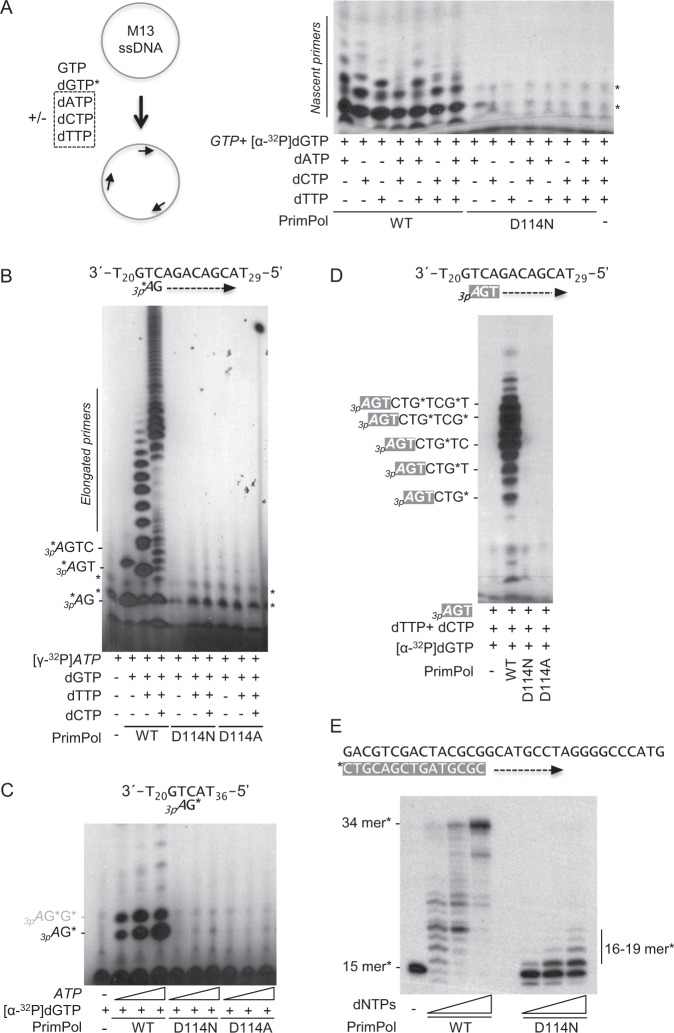
The PrimPol D114N mutation is deficient in primase activity. (**A**) WT, D114N, or D114A PrimPol and Pol γ are mixed with the M13 ssDNA plasmid. In the event that PrimPol primes the plasmid, Pol γ is able to extend the mature primer. The D114N mutant cannot form mature primers for Polγ extension. (**B**) The initiation and elongation of primers by PrimPol alone with limiting 5′ nucleotide. The D114N mutation is unable to initiate a primer compared to WT. (**C**) Dimer synthesis of PrimPol. Using the preferred priming sequence of 5′-GTCA-3′, the D114N PrimPol is unable to form the dinucleotide for primer initiation. (**D**) PrimPol extension of a supplied primer. The ability of the D114N mutant to extend a supplied primer with a 3′-triphosphate was assayed. Compared to the WT, the D114N mutant is unable to utilize the supplied 3′-triphosphate primer as a substrate. (**E**) Full elongation by PrimPol under standard polymerase conditions. When supplied with all dNTPs and a radiolabeled 15-mer annealed to a templating DNA, the WT is able to fully elongate the primer while the D114N mutant is able to catalyze a limited number of insertions.

With mounting evidence of the role of PrimPol as primarily a repriming enzyme^23,31,36–38^, we first examined the ability of PrimPol to synthesize primers, using a M13 ssDNA template to validate our hypothesis that the D114N mutation would hinder PrimPol catalysis (Fig. 3A). In comparison to wild-type PrimPol, we did not observe nascent primer production with the mutant in a heterogeneous sequence context. We then assessed D114N activity in a single template context [3′(T_20_)-G**TC**AGACAGCA-(T_29_)5′] by providing [γ-^32^P]ATP and the indicated dNTPs, to test sequentially the ability to initiate and elongate primers (Fig. 3B). Again, we observed a complete lack of primer initiation and the subsequent elongation by the D114N mutant, which parallels the null activity of a more drastic change of Asp^114^ to alanine (D114A in Fig. 2B^35^; Calvo *et al*., 2019). Next, to boost the formation of the initial dimer, we provided higher concentrations of the rate limiting nucleotide ATP and [α-^32^P]dGTP, on the template sequence 3′(T_20_)-G**TC**A-(T_36_)5′ (Fig. 3C). Even in the presence of high concentrations of the 5′ ATP nucleotide (100 µM), we failed to observe dimer formation using the D114N mutant protein. Although D114N PrimPol cannot form the initial dinucleotide for primer synthesis, it may retain the ability to elongate preexisting primers. To test that, we supplied the reaction with a synthetic 3-mer primer with a 5′-triphosphate, (3p*A*GT), which has previously been demonstrated to be important for the binding of PrimPol to the initiated primer^39^. In these conditions, extension of the primer was efficiently carried out by wild type PrimPol but not by the D114N mutant (Fig. 3D). Lastly, we examined the conventional DNA polymerase activity of the D114N PrimPol mutant, by using a mature 5′ radiolabeled ssDNA 15-mer (which is a valid primer despite the lack of the 5′ triphosphate) annealed to a ssDNA 34-mer. Although we observed a prominent reduction in polymerase activity, D114N still retained some ability to incorporate dNTPs onto a primer (Fig. 3E). Even taking into consideration that the D114N mutant incorporates nucleotides to lesser degree compared to wild-type, the residual catalytic activity was surprising in contrast to the complete loss of primase function.

### The D114N mutation retains catalytic activity but drastically reduces the kinetics of polymerization

**Figure 4:**
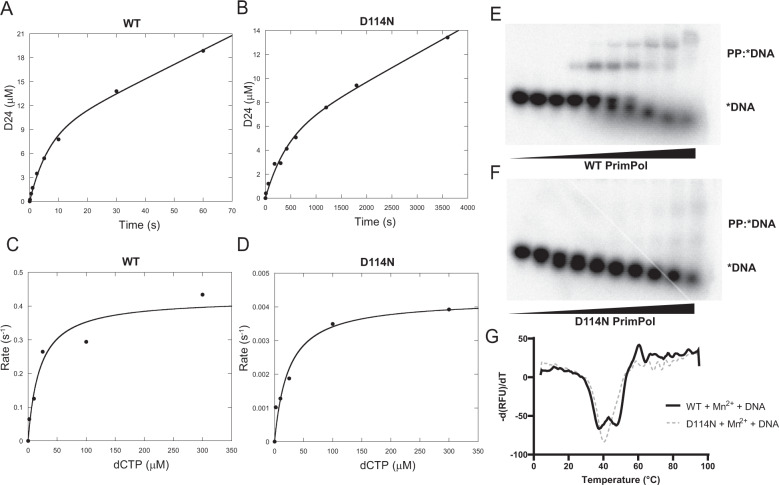
Kinetic and biochemical characterization of the PrimPol D114N mutation. (**A**) Pre-steady state burst kinetics of WT and (**B**) D114N PrimPol. The concentration of a 23-mer annealed to a 45-mer (DNA:DNA) was held in slight excess to protein and the incorporation of dCTP was plotted against time and fit to a burst equation. (**C**) *K*_d_ curves of WT and (**D**) D114N mutant PrimPol. Rates of dCTP incorporation were measured under single-turnover conditions where PrimPol is in excess of DNA:DNA and plotted against varying concentrations of incoming dCTP. (**E**) Electrophoretic mobility shift assays of WT and (**F**) D114N PrimPol with radiolabeled dsDNA substrates in the presence of Mn^2+^ where the upper bands correspond to the formation of the PrimPol:DNA complex. (**G**) Comparison of the stability of WT and D114N PrimPol by a thermal shift assay using SYPRO orange. The derivative of the melting curves were plotted and the local minima correspond to melting temperatures of the protein (see Fig. S3, Table S2).

In order to further probe the magnitude of the hindering D114N mutation on catalytic activity, we measured the kinetics of natural nucleotide incorporation under pre-steady-state burst and single-turnover conditions to compare the mutant and wild-type proteins. Under burst conditions where the DNA substrate is in slight excess of protein, there have been many examples of DNA polymerases exhibiting biphasic kinetics^40–42^. These kinetic observations indicate that in the overall kinetic mechanism of catalysis, product release of the DNA from the protein is slower in relation to the chemical catalysis of incorporation. We previously established that PrimPol exhibits a burst phase^28^, and we observed that the D114N mutation displayed similar biphasic kinetics (Fig. 4A,B). Compared to wild type, the burst rate of the mutant was approximately 60-fold slower, and the linear steady-state rate approximately 20-fold slower (Table 2).

**Table 2:** Summary of Pre-Steady State Kinetics of Wild-type (WT) and D114N PrimPol.

Burst			
	k_ss_ (s^−1^)	k_burst_ (s^−1^)	
WT	9.9 ×10^−3^ $\pm$ 4.8 ×10^−3^	1.5 ×10^−1^ $\pm$ 6.8 ×10^−2^	
D114N	4.7 ×10^−4^ $\pm$ 1.1 ×10^−4^	2.5 ×10^−3^ $\pm$ 5.6 ×10^−4^	
**Single Turnover**			
	**K**_**d**_**(μM)**	**k**_**pol**_ **(s**^**−1**^**)**	**k**_**pol**_**/k**_**d**_ **(μM**^**−1**^ **s**^**−1**^**)**
WT	19.7 $\pm$ 7.5	4.2 × 10^−1^ $\pm$ 4.2 ×10^−2^	2.1 × 10^−2^
D114N	23.9 $\pm$ 8.7	4.2 × 10^−3^ $\pm$ 4.2 ×10^−4^	1.8 × 10^−4^

Burst pre-steady state kinetic parameters for both WT and D114N PrimPol at at 25 °C were determined by fitting the time course data to the burst equation: [*product*] = *A*(1− *e*^−*k*^ *obs*^*t*^) + *A*(*k*_*ss*_) (*t*), where *A* is the burst phase amplitude, *k*_*obs*_ is the observed single exponential rate, *k*_*ss*_ is the steady-state rate, and *t* is the time. Single turnover kinetic parameters were determined by fitting the time course data with the following single exponential equation: [*product*] = *A*(1− *e*^−*k*^ *obs*^*t*^), where *A* is amplitude and *k*_*obs*_ is the observed single exponential rate, and *t* is the time. The single exponential rates were then plotted against each concentration of [dNTP] using a quadratic equation in order to extract the k_pol_, the maximal rate of incorporation, K_d_, the apparent binding constant for the incoming nucleotide, and k_pol_/K_d_, the overall efficiency for nucleotide incorporation. The errors represent the standard error values of the parameters that corresponds to a confidence level of 68.3%, or to one standard deviation.

To further examine the catalytic mechanism, we carried out single turnover experiments to calculate the k_pol_, the maximal rate of incorporation, K_d_, the apparent binding affinity of the incoming nucleotide, and k_pol_/K_d_, the overall incorporation efficiency (Fig. 4C,D). Single-turnover experiments provide a clearer comparison of the chemical catalysis steps that may be obscured by the linear phase under burst conditions. Importantly, the binding of the incoming nucleotide may also be compromised because the D114N mutant could potentially affect divalent metal coordination, which may appear as a change in the K_d_. Overall, we saw a 100-fold decrease in the overall incorporation efficiency (k_pol_/K_d_) with the mutant (Table 2). Examining the individual kinetic parameters that define the incorporation efficiency revealed that this 100-fold difference was reflected solely in the k_pol_, while the K_d_ for the incoming nucleotide remained nearly identical between the mutant and wild-type proteins. This drop of k_pol_ in D114N during polymerization activity explains perfectly its incapacity of primer synthesis.

### DNA binding ability and protein stability are hindered by the D114N mutation

We predicted that the D114N mutation was unlikely to have significant effects on DNA binding or overall structure, as described for a catalytically inactive mutation (D114A) of the same residue^35^; however, it could not be discarded that the change of Asp^114^ to Asn could have a significant effect on the stability of the whole protein, as described in other studies on PrimPol mutants^43^.

The binding of wild-type and mutant proteins to a template/primer DNA was compared through electrophoretic shift mobility assays (EMSAs). While the wild-type protein exhibited a similar DNA binding affinity to that described in previous work, a striking reduction in the DNA-binding capabilities of the mutant was evident (Fig. 4E,F). Taking into consideration that DNA binding and correct positioning of the template DNA is a prerequisite for nucleotide incorporation activity and that EMSAs may be unable to detect weak binding complexes^44^, we concluded that the D114N mutation diminished the DNA binding affinity of the protein but did not completely attenuate it. The reduction in activity or dsDNA binding may stem from the importance of Asp^114^ as a catalytic residue or a contributor to key interactions that coordinate with the DNA or stabilize the protein. In order to examine the latter effects of the mutation, we utilized differential scanning fluorimetry (DSF)^45,46^ to observe changes in the melting temperatures of the wild-type and mutant proteins as shown in Fig. 4. In comparison of the melting temperatures of wild-type to D114N PrimPol in the presence of dsDNA and Mn^2+^, we observed the presence of two melting temperatures in the wild-type protein and one in the mutant (Fig. 4G). The presence of the 47.17 °C ± 0.26 melting peak in the wild-type compared to the singular 40.17 °C ± 0.26 with D114N PrimPol appeared to indicate that the D114N mutation caused some instability within the protein (Table S2). In addition, due to the proximity of the melting temperature of the mutant to physiological temperature (37 °C), we conducted our kinetic assays at 25 °C.

We further analyzed if the two peaks found in wild-type PrimPol correspond to the polymerase domain (AA354) and the zinc finger domain (ZnF). We conducted the thermal shift assay with the isolated polymerase domain, the zinc finger domain^38^, or their combination in the presence of either Mg^2+^ or Mn^2+^ (Fig. S4A,B). The lower melting temperature peak appears to correspond to the ZnF and the more stable peak to the polymerase domain (Table S2). Taking this information into account in reference to the D114N mutant, the absence of the higher temperature minimum may represent the destabilization of the polymerase domain. Interestingly, further characterization demonstrates that both WT and D114N PrimPol were slightly more stable in the presence of Mg^2+^ than Mn^2+^ (Fig. S4C,D). Moreover, addition of dsDNA stabilized the wild-type PrimPol and Mn^2+^ complex, possibly by stabilizing the polymerase domain, while there was no effect on the Mg^2+^ complex (Fig. S4E,F). The dsDNA stabilization effect was notably absent in the mutant, which may be reflective of the reduced ability of the mutant to bind dsDNA as observed in the EMSAs in Fig. 4F (Fig. S4G,H).

### Respiratory capacity is ablated by reducing PrimPol levels

Because PrimPol was able to incorporate TFV-DP *in vitro*, although with a low efficiency, the role of PrimPol either mediating or protecting against mitochondrial toxicity remained uncertain. We predicted that PrimPol overexpression or knockdown cell lines could address the role of PrimPol in tenofovir-associated toxicity. If PrimPol had a protective effect against tenofovir toxicity, then the overexpression cell lines would fare better compared to the knockdown strains. Conversely, if PrimPol actively incorporates tenofovir and stalls replication, then the knockdown cell line would exhibit less phenotypes related to toxicity when treated with tenofovir. Due to the presence of tenofovir-caused nephrotoxicity in the renal proximal tubules of the kidney, we generated stable cell lines with overexpressed wild-type PrimPol or knocked-down levels of PrimPol using immortalized renal proximal tubular epithelial cells (RPTECs) (Fig. S5A)^47^.

Tenofovir treatment of cells can affect metabolism and reduce the respiratory capability of the mitochondria. Tenofovir is able to downregulate TRAP1, a regulator of glycolysis, which is accompanied by changes in cellular respiration^48^. As a first approach, we monitored TRAP1 levels through immunoblotting after treatment with tenofovir disoproxil fumarate (TDF) for 3 days to observe the potential of varying levels of TRAP1 as an indicator of corresponding changes in metabolism (Fig. S5B,C). We observed a downward trend in TRAP1 protein levels in both the scrambled and shRNA knockdown cell lines, but not in the overexpression strain. This result prompted us to further assess the potential of PrimPol to resist or enhance the effects of tenofovir treatment on metabolism through cellular respiration measurements using the Seahorse XF Analyzer (Fig. S5D)^49^.

**Figure 5:**
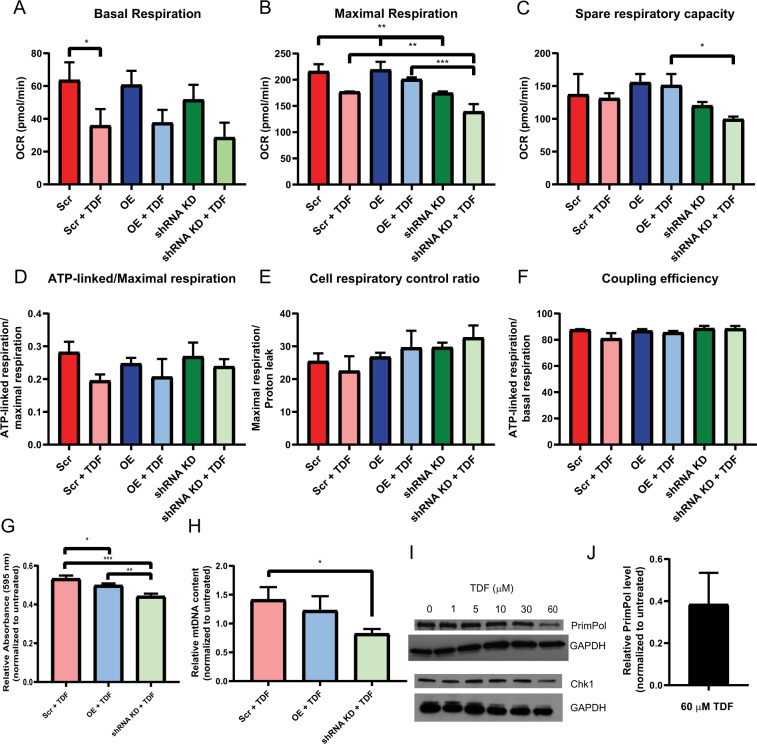
PrimPol knockdown RPTECs display reduced mitochondrial fitness and hypersensitivity to TDF treatment. Scrambled, PrimPol overexpression, and shRNA knockdown strains of RPTECs were treated with 30 µM TDF for 3 days and oxygen consumption rate was monitored (**A–F**). The spare respiratory capacity, (**C**), was calculated by subtracting the basal respiration from the maximal respiration. Internally normalized parameters were calculated to control for cell number (**D–F**). For toxicity experiments, RPTECs were treated with 30 µM TDF and parameters related to toxicity were measured after 5 days. (**G**) Cell proliferation was measured through the MTT assay. Absorbances of the treatment conditions were normalized to the untreated control. (**H**) Relative mtDNA content was quantified by qPCR and the treatment conditions were normalized to the mtDNA content of the untreated control. (**I**) Scrambled cells were treated with varying concentrations of TDF for 5 days and then immunoblotted for PrimPol. (**J**) The relative amounts of PrimPol at 60 µM in I) were quantified relative to the untreated control. Significance was determined by one-way ANOVA, n = 3. *p < 0.05, **p < 0.01, and ***p < 0.001.

Upon treatment with tenofovir, we observed a universal reduction in basal respiration and maximal respiration (Fig. 5A,B). Interestingly, even in the untreated controls, the shRNA knockdown RPTECs showed a lower maximal respiration rate compared to both scrambled and overexpression cell lines, which is recapitulated in the TDF treatment conditions. Consequently, there was a sharp reduction in the spare respiratory capacity, or the ability of the cell to respond to respiratory needs which may arise in stress conditions (Fig. 5C). In order to control for effects of respiration based on changes in cell number, we calculated the internally normalized parameters of ATP-linked respiration/maximal respiration, cell respiratory control ratio, and coupling efficiency (Fig. 5D–F). Of the three internally normalized parameters, the ATP-linked respiration to maximal respiration ratio displayed a downward shift with the treatment of TDF, and is thus the appropriate parameter to compare cell strains (Fig. 5D). Although there were significant decreases in basal and maximal respiration and spare respiratory capacity with the shRNA knockdown strains, the difference in the ATP-linked-respiration to maximal respiration ratio was absent, which suggests that the differences in cellular respiration were due primarily to decreases in cell count.

### PrimPol knockdown cells display increased sensitivity to tenofovir

In addition to examining the cellular respiration, it is essential to understand if PrimPol contributes to or alleviates mitochondrial toxicity in the context of tenofovir treatment. Cellular proliferation assays were utilized with the knowledge from the respiration experiments that suggested that the knockdown cell lines may be more sensitive to TDF treatment. After treatment of TDF for 5 days, cellular proliferation was monitored via the MTT assay and absorbances were normalized to untreated cells (Fig. 5G). Although a decrease in proliferation was observed in the overexpression cell lines compared to the scrambled strain, the shRNA knockdown RPTECs experienced a greater reduction in proliferation compared to both scrambled and overexpression cells. The apparent sensitivity of the shRNA knockdown cells to TDF treatment compared to the other cell lines corroborates our respiration data (Fig. 5A–D and S6A), implying that the reduction in cell number contributed to the decreased respiration. Altered mtDNA copy number is an additional phenotype related to NRTI-associated toxicity^8,50,51^. After cells were treated with TDF for 5 days, the relative mtDNA content was quantified using qPCR and normalized to untreated cells. In both the scrambled and overexpression cell lines, the amount of mtDNA was increased compared to the untreated cells (Figs. 5H, S7, and Table). In contrast, the PrimPol shRNA knockdown cells experienced a decrease in mtDNA compared to the untreated control, which may be an indicator of toxicity.

To validate our results, we observed the effect of the NRTI abacavir and efavirenz, a non-nucleoside reverse transcriptase inhibitor (NNRTI) on the proliferation of the RPTEC strains. In the case of abacavir, we expected that we would observe a similar decrease in proliferation in the knockdown strains. However, because abacavir is incorporated by PrimPol to a greater extent than tenofovir, the possibility that higher levels of PrimPol could mediate toxicity remained a possibility. We observed similar results to when the cells were treated with TDF in the proliferation assays, suggesting that PrimPol plays a protective role even in the case of ABC treatment (Fig. S6B). To confirm that our results are specific to NRTIs, we replicated the assays in the presence of efavirenz and observed no difference in proliferation between our overexpression and knockdown cell lines (Fig. S6C).

Consolidating both the cellular respiration and toxicity analyses provide the impression that the primary role of PrimPol in NRTI-associated toxicity is repriming downstream of chain-terminated nucleotides and preventing stalled replication forks. Given this putative role, we hypothesized that the cells may upregulate PrimPol levels in response to TDF treatment as a protective measure. Surprisingly, our findings were contrary to our predictions in that, under high doses of TDF (60 µM), we observed a downregulation of PrimPol protein levels in RPTECs that was similarly observed with Polγ (Fig. 5I,J)^48^. Recently, it was shown that PrimPol is upregulated by the activation of the ATR pathway^52^, perhaps through interactions with RPA^53,54^. It has also been previously described that NRTIs are able to downregulate Chk1, which is downstream of ATR^55^. Thus, TDF treatment may concurrently downregulate Chk1 and PrimPol. Indeed, immunoblotting for Chk1 also shows a decrease of protein levels under high dosing of TDF (Fig. 5I) and that Chk1 downregulation is associated with PrimPol downregulation. Although the full mechanism to which PrimPol and Polγ is downregulated with treatment of high concentrations of TDF is unclear, it is possible that decreased amounts of these proteins may contribute to manifestation of toxicity.

## Discussion

In this study we have identified and characterized the D114N *PRIMPOL* active site mutation found in an HIV+ patient with mitochondrial toxicity. The D114N mutation eliminates PrimPol primase activity, proven to be required for mitochondrial and nuclear DNA replication, and significantly attenuates its polymerase activity, whose role in the cell is unclear. We demonstrated that PrimPol incorporates the active form of tenofovir with a low efficiency compared to the natural nucleotide, making it somewhat unlikely that PrimPol directly contributes to TDF-induced mitochondrial toxicity. On the contrary, we suggest that PrimPol protects against tenofovir-caused toxicity by its repriming capabilities after replication has been stalled due to the incorporation of the chain terminator by Polγ or other potential mitochondrial polymerases^6,56^. This implies that loss of function mutations of PrimPol, such as D114N, in patients taking tenofovir-containing ART could predispose these individuals to mitochondrial toxicity.

### Structural basis for the loss of primase activity but residual polymerase activity in D114N PrimPol

It is vital to understand how mutations present in individuals may contribute to disease or in this particular case, how PrimPol mutations could influence the phenotype predisposing towards mitochondrial toxicity. Biochemical studies investigating how PrimPol variants function at a molecular level is beneficial not only in considering treatment options and strategies in patients, but also contributes to the basic mechanistic understanding of the enzyme. This study is the first to identify a PrimPol mutation in an individual in the context of HIV ART: a single G to A transition in the PrimPol gene implicated an Asp to Asn mutation at amino acid position 114. Asp^114^ is part of the invariant motif A (**D**x**E**), and is one of the 3 catalytic carboxylates forming the active site of AEP-like enzymes as PrimPols^21,22^. Mutation of any of these residues to alanine abolishes PrimPol activities^35^. Like the other two carboxylates, Asp^114^ is involved in metal coordination at the active site, which is critical for nucleotide incorporation.

Our initial hypothesis was that the D114N mutation would similarly compromise both primase and polymerase catalytic activities. However, we were surprised that PrimPol mutant D114N was able to retain some polymerase activity, but completely lacked primase activity. Some remaining activity can be explained as the substitution of a charged residue as Asp114 to Asn is more conservative than its change to an hydrophobic Ala. Kinetic analysis of the D114N mutant in DNA polymerase assays indicated a strong reduction in the k_pol_ which suggests a deficient metal coordination at the active site, which compromises (but still allows) nucleotide incorporation. On the other hand, a similar drop of k_pol_ in PrimPol D114N provokes a more dramatic consequence for dimer formation to start primer synthesis. This could be explained by the stricter and perhaps specific metal coordination required for binding the two initiating nucleotides. The crystal structure of PrimPol in priming mode with two catalytically competent metal ions would be critical in understanding the nuances of PrimPol activity when compared to the current structure^34^.

The extensive comparison of wild-type and D114N PrimPol through the thermal shift assay also revealed interesting phenomena. Although Mn^2+^ has been demonstrated to be crucial for priming activity, we observed that PrimPol tended to be more stable in the presence of Mg^2+^. Assuming that PrimPol may utilize either Mg^2+^ or Mn^2+^ in the cell^35,57^, the stabilization/destabilization effect may favor a particular mode for PrimPol. In agreement with this idea, a recent paper^58^ demonstrated that in the presence of Mn^2+^, a conformational transition step from non-productive to productive PrimPol:DNA complexes limits the enzymatic turnover, whereas, in the presence of Mg^2+^, the chemical step becomes rate limiting. The appearance of two melting minima for wild-type PrimPol, which correspond to the ZnF and the catalytic polymerase domain, may also aid in understanding the primase mechanism of PrimPol. Because there are two distinct peaks for each domain, it is possible that the two domains may be able to function somewhat independently from one another at a structural level. It is worth mentioning that PrimPol lacking the ZnF is polymerase competent but is not able to start primer synthesis^23,38^. The lack of two melting temperature minima for D114N PrimPol reveals that the mutation destabilizes the polymerase domain. Interestingly, analysis of the Y89D mutation in PrimPol shows a similar destabilization^43^. To discern whether the single amino acid mutation causes the destabilization of the catalytic domain, or if coordination of a metal divalent ion assists in stabilization of the protein would require further study.

It is noteworthy that the patient identified with the D114N PrimPol mutation was heterozygous for the mutation. It may be interesting to determine if partially reducing the total amount of functional PrimPol is substantial enough to predispose a patient to off-target toxicity, considering the knockdown of PrimPol in our cell lines was highly efficient. In addition, because many factors could contribute to the complex toxicity caused by NRTIs, it is difficult to attribute this patient’s toxicity solely to this active site mutation. The potential for other polymerases or other molecules involved in oxidative stress or maintaining dNTP pools to contribute to mitochondrial toxicity may explain why we do not observe other mutations in PrimPol in our patient cohort^19^.

### Nucleotide sequence context can influence NRTI incorporation by PrimPol and other polymerases

Upon testing the “preceding nucleotide” effect on TFV-DP incorporation by PrimPol, we did not expect the striking differences observed. Although there are numerous examples of enzymes with nucleic acid sequence preferences^59–61^, whether the current observations might extend to other polymerases requires further investigation. Possible explanations for this effect may be due to additional binding interactions of TFV-DP with PreT, or the PreT dsDNA substrate may have an altered nucleic acid structure^62^ that may affect the active site conformation and subsequently TFV-DP incorporation that may be related to the acyclic nature of the chemical structure^63^. Determining the ternary structure of PrimPol:dsDNA:TFV-DP would be valuable to reveal the mechanism behind this nucleotide preference.

Understanding the biochemical and structural mechanism(s) underlying such a preference for inserting TFV-DP next to a thymine at the primer terminus could prove to be useful in drug design efforts^64^. For example, if a nucleoside analog inhibitor was being developed as an antiviral therapy, an important consideration would be avoidance of off-target effects for host polymerases such as Polγ. Based upon our findings of the influence of sequence specificity this assessment should be carried out using a variety of DNA substrates. While we have confirmed the preceding nucleotide effect in the specific case of PrimPol and TFV-DP, it may be crucial to extend these experiments to other NRTIs and the respective target and host polymerases. In our experiments, we were able to observe discrimination differences up to 10-fold with different nucleotides in the n-1 position (Table 1). Thus, it is highly likely that NRTI discrimination values in the current literature may be under- or overestimated. Assessing the potential for sequence effects on NRTI incorporation by Polγ would be essential to provide more accurate estimates of the contribution of the enzyme to NRTI-associated toxicity.

### PrimPol plays a key role in protection against tenofovir-associated toxicity

In the present study, we addressed the possibility that PrimPol can alleviate tenofovir-associated toxicity. In light of our cellular experiments, we propose that the benefits of the repriming ability outweighs any possible toxicity due to tenofovir incorporation by PrimPol. One caveat to our experimental setup was that the RPTECs were treated with TDF for a short amount of time (3–5 days). However, if nephrotoxicity arises in patients after steady, low, long-term exposure to antiretroviral therapies, then the possibility that PrimPol could incorporate tenofovir at a low level over years of treatment still exists. Thus, it would be desirable to recapitulate our assays under longer treatment periods of TDF to more appropriately mirror a clinical situation.

Intriguingly, we observed decreased amounts of PrimPol when cells were treated with a high amount of TDF. A previous study demonstrated that treating cells with a high amount of TFV decreased the protein levels of Polγ and the authors suggest that the downregulation of Polγ may lead to toxicity^48^. While it is unknown if PrimPol and Polγ cause toxicity through downregulation or are downregulated as a result of toxicity, PrimPol appears to be regulated by a similar pathway as Polγ. In light of recent findings showing that PrimPol is regulated in part by the ATR pathway, we also determined that PrimPol regulation by TDF is associated with Chk1 regulation. Our current findings support these previous studies and suggest a potential pathway to investigate to determine the mechanism of regulation of PrimPol by tenofovir. It will also be of value to examine the levels of Polγ and PrimPol in our cohort of patients to identify any changes in proteins level that may stem from long-term exposure to NRTIs in antiviral therapy.

### Concluding remarks

In conclusion, we established the role of PrimPol in NRTI-associated toxicity and identified the PrimPol-inactivating D114N mutation in an HIV+ patient experiencing toxicity under a tenofovir-containing regimen. Cells with the primase-deficient D114N are expected to behave as the PrimPol shRNA knockdown strains, which display reduced mitochondrial respiration and increased sensitivity to toxic side-effects of tenofovir treatment. This hypothesis must be further validated by examining the impact of the D114N on mitochondrial fitness and toxicity at a cellular level. While we have characterized a single mutation in this study, other mutations of PrimPol in other disease contexts such as myopia or adenocarcinomas have been identified that could be further explored^38,65–67^.

## Materials and Methods

### Protein Purification of WT, D114N, AA354, and ZnF PrimPol

PrimPol WT, D114N, isolated polymerase domain (AA354), and isolated zinc finger domain (ZnF) were purified following a modified protocol of^28^. The pET28a-PRIMPOL expression vector was transformed into *E. coli* BL21(DE3)-pRIL cells. For 1 L of LB + kanamycin, 10 mL of overnight culture was added for protein production. Cells were grown at 37 °C until the OD_600_ 0.6, at which point the flasks were chilled at 4 °C, PrimPol induced with 1 mM IPTG, and allowed to induce overnight at 19 °C (approximately 16 hours). Cells were harvested at 12,000 × g for 15 min at 4 °C, and the pellet flash frozen in liquid nitrogen and stored at −80 °C. For every 1.5 g of pellet, 5–10 mL of lysis buffer (Buffer A: 50 mM Tris-HCl pH 8, 1 M NaCl, 10 mM Imidazole, 10% v/v glycerol, EDTA-free protease inhibitor, 0.5 mM TCEP, 0.1% Triton X-100). The suspension of cells was lysed by passing the cells through a high-pressure homogenizer (Emulsiflex) 2–3 times. The lysate was centrifuged at 28,000 × g for 1 hour at 4 °C. The supernatant was loaded onto a 5 mL HisTrap FF crude column equilibrated in Buffer A and washed with buffer A after protein loading until the A_280_ was stabilized. The column was then equilibrated with buffer B (Buffer B: 50 mM Tris-HCl pH 8, 50 mM NaCl, 10% v/v glycerol, 0.5 mM TCEP) to reduce the salt concentration. The column was washed with 5 CV 95% Buffer B and 5% Buffer C (Buffer C: 50 mM Tris-HCl pH 8, 50 mM NaCl, 600 mM imidazole, 10% v/v glycerol, 0.5 mM TCEP) or until A_280_ was stabilized. The protein was then eluted by a 0–100% gradient of buffer B and buffer C, separating the elution into 0.5 mL fractions across 5 column volumes (25 mL). The fractions were resolved by 4–20% Tris-glycine gel in SDS and proteins stained by Coomassie blue staining. The cleanest fractions were pooled and TEV protease was added to the pooled fractions (1:10 TEV:total protein w/w). The solution was dialyzed with buffer D (Buffer D: 50 mM Tris-HCl pH 8.0, 300 mM NaCl, 10% (v/v) glycerol, 0.5 mM TCEP) overnight at 4 °C using 25 kD dialysis membrane tubing. The protein was loaded onto a 5 mL HisTrap FF column equilibrated in Buffer B. Buffer B was used to wash the column until the A_280_ was stabilized. 5% buffer C was used to wash the column and then a 0–100% gradient of Buffer B and Buffer C was used in the same manner as the previous column. Because the His-tag was cleaved with TEV protease, PrimPol eluted in the 5% imidazole wash, although we did observe the presence of cleaved PrimPol in the 0–100% gradient fractions. The clean fractions were pooled, concentrated, and buffer exchanged to about 0.5–2 mL into buffer E (Buffer E: 50 mM Tris-HCl pH 7.5, 300 mM NaCl, 5% (v/v) glycerol, 0.5 mM TCEP) using a 10 K centrifugal filter. The protein was then separated using a Superdex 200 Increase 10/300 size exclusion column using Buffer E. Clean fractions were pooled, concentrated, aliquoted, and flash frozen and stored at −80 °C.

### Oligonucleotide labeling and annealing

In general, primer oligonucleotides were labeled at the 5′ end with [γ−32P]ATP and T4-PNK with the provided reaction buffer for 30–45 minutes at 37 °C. The reaction was then stopped by heat shock for 5 minutes at 70–95 °C. For the primase assays, the labeled oligos and unlabeled template were mixed at a 1:2 ratio in 50 mM Tris-HCl pH 7.5, 300 mM NaCl and heated for 10 minutes at 80 °C, and slowly cooled down to room temperature. In all other kinetic assays, primer and template were mixed in a 1:1.1 ratio in 10 mM Bis-Tris Propane, pH 7.0, 300 mM NaCl and annealed by heating to 90 °C for 5 minutes, 55 °C for 15 minutes, and 37 °C for 10 minutes.

### Burst and single turnover kinetics

Kinetic assays to measure the activity of WT and D114N PrimPol were based on the single incorporation of dCTP on a radiolabeled DNA primer (5′-GCCTCGCAGCCGTCCAACCAACT-3′) annealed to a DNA template (5′-GGACGGCATTGGATCGAGGTTGAGTTGGTTGGACGGCTGCGAGGC-3′).

Burst and single turnover kinetics were conducted as previously done, with modifications to the protocol noted below^28^. All kinetics assays were carried out using reaction buffer (10 mM Bis-Tris Propane, pH 7.0, 300 mM NaCl) as reported previously. Briefly, PrimPol was incubated with the primer:template and mixed with dCTP and 10 mM MnCl_2_ before quenching with 0.5 M EDTA. Products were collected in a tube with formamide dye [0.1% bromophenol blue (w/v), 0.1% xylene cyanol (w/v)] and separated by denaturing urea PAGE. The radiolabeled products were visualized by the Molecular Imager FX phosphorimager (Bio-Rad) and quantified by Quantity One, version 4.6.9 (Bio-Rad).

In the case of measuring the kinetics of wild-type PrimPol, pre-steady state kinetic assays were performed using the RQF-3 rapid chemical quench apparatus (KinTek) at room temperature. The incorporation of the D114N variant was slow enough to allow for manual mixing reactions to be employed. For burst reactions, 10 µM WT or D114N PrimPol was incubated with 30 µM annealed primer:template before mixing with 200 µM dCTP and 10 mM MnCl_2_ at 37 °C. The [product] was plotted against time and the data points were fit to a burst equation, [*product*] = *A*(1− *e*^−*k*^ *obs*^*t*^) + *A*(*k*_*ss*_) (*t*), where A is the burst phase amplitude, k_obs_ is the observed single exponential rate, k_ss_ is the steady-state rate, and t is the time.

For single turnover experiments, 10 µM WT or D114N PrimPol was mixed with 300 nM primer:template and mixed with 0–300 µM dCTP and 10 mM MnCl_2_. Reactions for the comparison of D114N to WT were done at room temperature due to relative protein instability at 37 °C. The single data points were fit to a single turnover equation, [*product*] = *A*(1−*e*^−*k*^ *obs*^*t*^), where A is amplitude and k_obs_ is the observed single exponential rate, and t is the time using Kaleidagraph. The rates were then plotted against [dCTP] used and fit to a quadratic equation, [*dNTP*] = 0.5 (*K*_*d*_ + [*dNTP*] + [*k*_*pol*_])− 0.5{(*K*_*d*_ + [*dNTP*] + [*k*_*pol*_])^2^ − 4[*dNTP*] [*k*_*pol*_]}^1/2^, in order to extract the k_pol_, the maximal rate of incorporation, K_d_, the apparent binding constant for the incoming nucleotide, and k_pol_/K_d_, the overall efficiency for nucleotide incorporation^28^. In both the burst and single turnover experiments, the values represent the fit estimate for the parameter ± one standard deviation.

For tenofovir diphosphate incorporation assays, the 3′-end of the primers were varied, D20A and D45: 5′-GCCTCGCAGCCGTCCAACCX_1_–3′, where X_1_ is A, C, G, or T. The corresponding annealed oligo templates were 5′-GGACGGCATTGGATCGAGGTTGAGTX_2_GGTTGGACGGCTGCGAGGC-3′, where X_2_ is the natural base pair to X_1_. Initial experiments used 200 µM TFV-DP under single turnover conditions, monitoring TFV-DP incorporation at 0, 2, 5, 30, 60, and 120 minutes. Then experiments were done under single turnover conditions using 0–1000 µM TFV-DP, and control dATP experiments used 0–100 µM. K_d_ curves were generated from each of these rates using a quadratic equation to estimate the k_pol_ and K_d_. In the case of TFV-DP, the rates did not vary with [TFV-DP], so k_pol_ was calculated by taking the average of all rates. The amplitude, however, did vary with TFV-DP concentration, so the K_d_ was calculated by plotting the amplitudes against [TFV-DP].

Confirmation experiments to validate the preceding nucleotide preference used a different oligo: D21A and D36, 5′-TCAGGTCCCTGTTCGGGCGCX_1_–3′ and 5′-TCTCTAGCAGTX_2_GCGCCCGAACAGGGACCTGAAAGC-3′, using 200 µM TFV-DP under single turnover experiments at 0, 2, 5, 30, 60, and 120 minutes. Additional experiments to observe if the preference was present with the zinc-finger knockout (amino acids 1–354) used 10 µM PrimPol1–354 and the D20A:D45 substrates at 0, 2, 5, 30, 60, and 120 minutes. Additional experiments were carried out to observe the preference effects using 200 µM d4T-TP, (−)-3TC-TP, or (−)-FTC-TP. For d4T-TP incorporation experiments, D22T:D45 was used: 5′-GCCTCGCAGCCGTCCAACCAAX_1_–3′ and 5′- GGACGGCATTGGATCGAGGTTGAX_2_TTGGTTGGACGGCTGCGAGGC. For (−)-3TC-TP and (−)-FTC-TP experiments, D23C:D45 was used: 5′-GCCTCGCAGCCGTCCAACCAACX_1_–3′ and 5′-GGACGGCATTGGATCGAGGTTGX_2_GTTGGTTGGACGGCTGCGAGGC-3′. The reactions were quenched for these experiments at 0, 30 seconds, 1, 3, 10, 30, and 120 minutes.

### Study participants and procedures

Study participants were enrolled at the Yale-New Haven Hospital from April 2011 to March 2013. The details of the study design for this cohort have been described previously^33^. In brief, for this PrimPol sub-study, cases (n = 13) comprised HIV-infected individuals on ART for at least 12 months with clinical and/or laboratory toxicities associated with mitochondrial toxicity. Cases were matched by age, sex, and race/ethnicity to HIV-negative controls (n = 19). All participants gave their written informed consent before participation in the study. The study protocol was approved by the Institutional Review Board of the Yale School of Medicine and all the research was performed in accordance to the relevant guidelines and regulations.

At study enrollment, participants answered a brief survey comprised of demographic characteristics and past medical history. Medical records of HIV-infected participants were reviewed, and disease characteristics and laboratory data (complete blood count, serum chemistries, liver function test, lipid profile, urinalysis, HIV RNA copy number, and CD4 + T-cell count) were extracted. Each participant gave about 20 ml of venous blood at the time of enrollment. Peripheral blood mononuclear cells (PBMCs) were isolated from whole blood within 2 hours of collection using Ficoll gradient (Ficoll-Hypaque; ICN) as described previously^68^. Aliquots of PBMCs were stored at −80 °C until DNA extraction for the experiments.

### DNA extraction and sequencing

Genomic DNA was extracted from PBMCs using TRIzol Reagent (Invitrogen, Carlsbad, CA, Cat.No.15596026) according to the manufacturer’s instructions. This sub-study included only study participants with sufficient archived DNA for the analysis (cases, n = 13, and controls, n = 19). Conversed active site and zinc finger coding exons were amplified with target specific primers (3th exon forward primer: 5′-TGGGCAACAGAGCTGACTC-3′, 3th exon reverse primer: 5′-GAAAAACTTGAGTTGGCCATT-3′; 5th exon forward primer: 5′-TAAGATGCGGTGTGTGGAGA-3′, 5th exon reverse primer: 5′-CGGTCTGATGGAGAAAGCTG-3′; 9th exon forward primer: 5′-GTGAATAAAGATGGCATTAAAGGAGG-3′, 9th exon reverse primer: 5′-ATTTTTAAAACAAAATAGTTTTCATATTCGCAAC-3′). All primers were synthesized from Keck biotechnology resource laboratory of Yale university. PCR products were collected using QIAquick gel extraction kit (Qiagen,Germany, Cat.No.28704) according to the manufacturer’s instructions. Samples were sent to Keck biotechnology resource laboratory of Yale university for further sequencing. SnapGene Viewer was used for sequence alignment.

### Site-directed Mutagenesis of D114N and D114A

The wild-type construct was previously subcloned into a pet28a vector^28^. A TEV-cleavage site was introduced using the megaprimer method to replace the FKBP12 protein in an N-terminal His-tag-TEV-FKBP12 expression construct^69^. Site directed mutagenesis to introduce the D114N mutation was carried out using the New England Biolabs Q5 Site-Directed Mutagenesis Kit using the forward and reverse primers 5′-GTGTGCAAGCTTTATTTTAACTTGGAATTTAACAAACC-3′ and 5′-GGTTTGTTAAATTCCAAGTTAAAATAAAGCTTGCACAC-3′. The PCR products were transformed into *E. coli* XL10-Gold cells and then the isolated plasmid DNA was sequenced to confirm successful cloning or mutagenesis.

The plasmid pET16::*CCDC111* containing the gene coding for WT PrimPol was used as template to generate the D114A mutation by the QuikChange Site-Directed Mutagenesis protocol (Stratagene). Oligonucleotides used to introduce the mutation were synthesized by Sigma Aldrich (St Louis, MO, USA): D114A-sense 5′ GTGCAAGCTTTATTTTGCTTTGGAATTTAACAAACCTGCCAACCC 3′ and D114A-antisense 5′ GGGTTGGCAGGTTTGTTAAATTCCAAAGCAAAATAAAGCTTGCAC 3′. The specific D114A mutation and the absence of other mutations in the *PRIMPOL* gene was confirmed by sequencing the recombinant plasmid that was kept in *E. coli* DH5α.

### Protein Purification of D114A PrimPol

Note that the protocol for D114A varies slightly to the WT and D114N purification as described in these current methods. PrimPol D114A protein expression and purification was performed identically to the WT PrimPol^21^ in *E. coli* BL21(DE3)-pRIL cells were transformed with the expression plasmid pET16::*CCDC111-*D114A. An overnight culture (50 ml) was incubated at 37 °C and used to inoculate 2 L of LB + ampicillin and chloramphenicol. Cells were grown at 30 °C up to O.D._600_ 0.8, and then PrimPol production was induced with 1 mM IPTG (Ref. 367–93–1, Sigma Aldrich, St Louis, MO, USA) for 2,5 h. Cells were harvested at 12,000 g for 5 min at 4 °C, and the resulting bacterial pellet (≈3 g/L) was frozen at −20 °C. Approximately 6 g of cells were resuspended in 100 mL lysis buffer (Buffer A: 50 mM Tris-HCl pH 8, 1 M NaCl, 10% glycerol, 1 mM PMSF, 2 mM β-mercaptoethanol, 10 mM imidazole and 400 mM AcNH_4_) and lysed by 10 min sonication pulses. The lysate was centrifuged at 27,000 g for 30 min at 4 °C to remove cell debris. Supernatant was incubated in batch with 2 mL HisPur Ni-NTA Resin (Ref. 88222; Thermo Scientific, Waltham, MA, USA) during 2 h at 4 °C. Resin was washed in batch with Buffer A and packed into a column. The resin was washed with 40 CV (column volume) of Buffer A containing 20 mM imidazole. Then washed with Buffer B (50 mM Tris-HCl pH 8, 10% glycerol, 1 mM PMSF, 2 mM β-mercaptoethanol) and 1 M NaCl, and later on with Buffer B and 50 mM NaCl. Finally, the protein was eluted with buffer B supplemented with 50 mM NaCl and 200 mM imidazole. Fractions containing PrimPol were loaded in a Heparin Sepharose 6 Fast Flow (Ref. 17–0998 from GE Healthcare, Chicago, IL, USA), washed with 10 CV of Buffer B with 50 mM NaCl, then with 10 CV of Buffer B with 100 mM NaCl, and finally eluted with Buffer B and 1 M NaCl. Fractions containing 99% pure PrimPol were dialysed in a Slide-A-Lyzer Dialysis Cassette (Ref. 66380 from Thermo Scientific, Waltham, MA, USA) against Buffer C (25 mM Tris-HCl pH 8, 50% glycerol, 500 mM NaCl and 1 mM DTT) for at least 2 h at 4 °C and stored at −20 °C.

### Full extension polymerase assay on a specific primer:template molecule

Full extension of a specific sequence substrate used a primer: 5′-CTGCAGCTGATGCGC-3′ and template: 5′ -GTACCCGGGGATCCGTACGGCGCATCAGCTGCAG-3′ (in a 1:2 ratio). Reaction mixtures (in 20 µL) contained Buffer R [50 mM Tris–HCl pH 7.5, 40 mM NaCl, 2.5% (w/v) glycerol, 1 mM DTT, 0.1 mg/mL BSA, 1 mM MnCl_2_], 2.5 nM [γ-^32^P]-labeled primer:template, 200 nM purified PrimPol and dNTPs (1, 10 100 µM). Reactions were incubated during 30 min at 30 °C, and stopped by adding 8 μL of formamide loading buffer, then loaded onto 8 M urea-containing 20% polyacrylamide sequencing gels of 30 cm long and run 2 h at 30 W. Following denaturing electrophoresis, primer extension was detected by autoradiography using AGFA CP-BU NEW Healthcare NV Medical X-RAY films blue (Ref. EWPKK, Mortsel, Belgium) and developed by a Kodak X-OMAT 2000 Processor (Rochester, NY, USA).

### Primase assay using M13mp18 template

Single-stranded M13mp18 DNA (5 nM) was used as template to assess priming activity of PrimPol (400 nM) in the presence of indicated dNTPs (10 µM) and 16 nM [α-^32^P]dGTP (250 µCi; 3000 Ci/mmol). Reaction mixtures (20 µL) in Buffer R, were incubated 30 min at 30 °C then stopped by adding 8 μL of formamide loading buffer, and loaded onto 8 M urea-containing 20% polyacrylamide sequencing gels (60 cm) and run 2 h at 50 W. After electrophoresis, products were detected by autoradiography using AGFA CP-BU NEW Healthcare NV Medical X-RAY films blue (Ref. EWPKK, Mortsel, Belgium) and developed by a Kodak X-OMAT 2000 Processor (Rochester, NY, USA).

### Primase assay on specific oligonucleotide templates

Primase assays were carried out using the following unlabeled ssDNA oligonucleotides as templates: 3′-(T)_20_G**TC**C(T)_36_–5′ or 3′-(T)_20_G**TC**AGACAGCA(T)_29_–5′. The reaction mixture (20 µL) in Buffer R contained 1 µM ssDNA template, 400 nM PrimPol, 16 nM [γ-^32^P]*ATP* or [α-^32^P]dGTP and indicated dNTPs at 10 µM. Dimer synthesis experiment was measured using *ATP* as a 5′nucleotide (1, 10, 100 µM). Pre-made _3P_*A*GT primer (10 µM) (synthesized by IDT, Coralville, IA, USA) was used to measure the elongation capacity of PrimPol variants. After an incubation time of 30 min at 30 °C, reactions were stopped adding 8 μL of formamide loading buffer. Synthesized primers were resolved in a 8 M urea-containing 20% polyacrylamide sequencing gels (60 cm) and run 2 h at 50 W. After electrophoresis, products were detected by autoradiography using AGFA CP-BU NEW Healthcare NV Medical X-RAY films blue (Ref. EWPKK, Mortsel, Belgium) and developed by a Kodak X-OMAT 2000 Processor (Rochester, NY, USA)..

### Differential scanning fluorimetry (Thermal shift assay)

The thermal shift assay was carried out using SYPRO orange dye to monitor protein unfolding. 5 µM PrimPol WT, D114N, AA354, or ZnF alone or in combination with each other, were mixed with additional combinations of 5 µM dsDNA (D20A/D45), and 10 µM MgCl_2_ or MnCl_2_ in 50 mM Tris pH 7.5, 300 mM NaCl, 0.5 µM TCEP, 5% glycerol, and a final concentration of 5x SYPRO orange dye in a 96-well PCR plate. The plates were placed in a BioRad CFX connect real time system and held at 4 °C for 5 minutes, then raised to 95 °C by 0.5 °C steps every 30 seconds. At each 30 second step, the relative fluorescence units were measured using the FAM channel. The derivatives of the melting curves were obtained from the Bio-Rad CFX Manager software and plotted against temperature. Each qPCR plate contained three (combined zinc finger and polymerase) or six (full length protein) technical replicates and to determine the melting temperatures, each value was averaged and the standard deviation was calculated. For the full length PrimPol thermal shift assays, two biological replicates were conducted.

### Cell culture maintenance and reagents

Immortalized renal proximal tubular epithelial cells (RPTECs) stably transfected with the OAT1 receptor were acquired from ATCC. RPTECs were maintained in DMEM:F-12 (containing 2.5 mM L-glutamine, 15 mM HEPES, 0.5 mM sodium pyruvate, and 1200 mg/L sodium bicarbonate) supplemented with 0.3 µg/mL puromycin, 100 µg/mL G418, 25 ng/mL PGE1, 3 pg/mL triiodothyronine, 25 ng/mL hydrocortisone, 10 ng/mL hEGF, 3.5 ug/mL ascorbic acid, ten-fold diluted ITS-G (10x stock), and 5% HI-FBS. After transduction with lentivirus containing PrimPol shRNA or overexpression plasmids, 200 µg/mL hygromycin was supplemented to the media. Cells were passaged every 3 days by a 1:10 split.

### Cell line transfection and transduction

HEK293T cells were cultured in [add media here] and transfected with plasmids containing dR8.91, VsV-G, and a third with either the shRNA knockdown (pLKO.1) or overexpression construct (pLenti). Briefly, HEK293Ts were seeded to 70–80% confluency and allowed to adhere to a 6-well plate. A ratio of 1:0.1:1 (0.75 µg dR8.01, 75 ng VsV-G, 0.750 µg pLKO.1/pLenti) were combined in a tube. Approximately 1 mL of Serum-free [DMEM] was mixed with 4.7 µg PEI (3:1 PEI:total DNA ratio) and incubated at room temperature for 5 minutes. The media:PEI mixture was added dropwise to the three-plasmid solution and incubated for 15 minutes at room temperature. The final mixture was then added to HEK293T cells. After 24 hours, the media was replaced with complete growth media for RPTECs (DMEM/F12). After another 24 hours, the media containing the lentivirus was aliquoted into 1 mL volumes and flash frozen and stored at −80 °C until use.

For transduction of RPTECs, 6-well plates were plated to approximately 40–50% confluency on the day of transduction. The virus was thawed and added to the adherent cells at 37 °C overnight. In some cases, the virus was diluted 2-fold with complete growth media. The media was replaced the next day with complete growth media for 24 hours at 37 °C. The following day, the media was replaced with selection growth media containing 200 µg/mL hygromycin. After seven days, the cells were immunoblotted for the overexpression or knockdown of PrimPol to confirm successful transduction.

### Cell counting

6-well plates were seeded with 50,000 cells and allowed to adhere. On the second and fourth days, cells were trypsinized using 0.25% trypsin-EDTA and counted using trypan blue and the Countess II Automated Cell Counter (ThermoFisher). The cell counts were plotted against time after seeding. Each well containing cells was counted three times and each cell type consisted of three biological replicates.

### Immunoblotting

TRAP1, PrimPol, or Chk1 levels in response to TDF treatment were measured through immunoblotting. 6-well plates were plated with 100,000 cells and allowed to adhere overnight. After treating RPTECs with 30 µM TDF for TRAP1 for 3 days or 1–60 µM TDF for 5 days for PrimPol, cells were harvested by washing with cold PBS two times followed by the addition of RIPA buffer supplemented with protease inhibitor (Roche) and cell scraping. Cells were incubated at 4 °C on a tube shaker for 30 minutes and centrifuged at 18,000xg for 10 minutes at 4 °C. The supernatant was collected and protein levels were measured through the BCA assay to normalize total protein loading. 10–30 µg of total protein were loaded onto a 4–20% Tris-Glycine gel and run in SDS buffer at 200V for 35 minutes. The loaded protein was transferred to a nitrocellulose membrane using the iBlot2 transfer system. The membrane was blocked with 5% milk for one hour, incubated with primary antibody overnight at 4 °C (1:1000 rabbit anti-TRAP1, 1:1000 rabbit anti-Chk1, 1:1000 rabbit anti-GAPDH, 1:500 rabbit anti-PrimPol), washed with 1x TBST three times for 5 minutes each, incubated with secondary antibody (anti-rabbit IGG, HRP-linked 1:1000) for 1 hour at room temperature, and washed with 1x TBST three times for 5 minutes each. The membranes were exposed to enhanced chemiluminescence reagent for TRAP1, Chk1, and GAPDH or SuperSignal West Femto Maximum Sensitivity Substrate for PrimPol for 1 minute. The membranes were then exposed to film for 5 seconds to 5 minutes and developed. Blots were quantified using ImageJ. In order to normalize across blots, the TRAP1/GAPDH or PrimPol/GAPDH levels of the treatment conditions were further normalized to the untreated control for each cell line, n = 3. Significance was determined by one-way ANOVA using GraphPad Prism. *p < 0.05, **p < 0.01, and ***p < 0.001.

### Cell proliferation assays

5000 cells were plated into 96-well plates and allowed to adhere overnight. On the next day, 30 µM tenofovir disoproxil fumarate (TDF), 100 µM abacavir (ABC), or 20 µM efavirenz in complete media was used to replace the media. On the third day of treatment, the media was replaced with fresh media with TDF. On the fifth day, a solution of 6 mM MTT in PBS was diluted to 1 mM in growth media and 100 µL of the solution was added to each well. After 3 hours of incubation at 37 °C, 150 µL of stop solution was added to each well (10% H_2_O v/v, 4% NP-40 v/v, 0.34% concentrated HCl v/v in isopropanol). The plates were protected from light and were shaken overnight at room temperature. The well solutions were resuspended by pipetting and absorbances were read at 590 nm. The absorbances were then subtracted from the background (no cells) and then normalized to the untreated cells. For each biological replicate (one 96-well plate), six technical replicates were done (6 wells in each plate). Significance was determined by one-way ANOVA using GraphPad Prism, n = 3. *p < 0.05, **p < 0.01, and ***p < 0.001.

### mtDNA quantification

100000 cells were seeded into 6-well plates and allowed to adhere overnight. The cells were then treated with 30 µM TDF for 5 days, with replacement of media on the third day. On the fifth day, the cells were detached with 0.25% trypsin-EDTA for 5 minutes and pelleted at 150xg for 5 minutes. The pellet was resuspended with PBS and pelleted an additional two times and stored at −80 °C until lysis for DNA harvesting. Total DNA was then isolated from the cells using the Qiagen DNeasy Blood & Tissue Kit, lysing the cells by adding PBS and vortexing for at least 30 seconds. The DNA concentrations were quantified spectrophotometrically and then diluted to 3 ng/µL with sterile H_2_O.

Quantification of mtDNA was followed using a qPCR-based method according to^70^. Each well in a 96-well plate contained 25 µL of reaction mixture: 2 µL of 3 ng/µL DNA (6 ng total), 2 µL of 400 nM qPCR primer pair (32 nM final), 12.5 µL of SYBR Green Master Mix, and 8.5 µL of nuclease-free water. The qPCR primer pairs target either the mitochondrial tRNA-Leu(UUR) gene or the nuclear B2-microglobulin gene. The qPCR reaction mixtures were heated to 95 °C for 3 minutes, then went through 50 cycles of 95 °C for 10 seconds and 60° for 30 seconds (reading the RFU at the end of each cycle using the SYBR channel), and finally 95 °C for 10 seconds, and then ramping up from 65 °C to 95 °C in 0.5° increments every 5 seconds (reading the RFU at every 5 seconds). After obtaining the C_T_ values for both mtDNA and nucDNA from the Bio-Rad CFX Manager software, the relative amount of mitochondrial DNA content was calculated by 2 ×2^ΔCT^, where ΔC_T_ is nucDNA C_T_ – mtDNA C_T_. The relative mtDNA content values were then normalized to the untreated control. For each biological replicate (one qPCR plate), three technical replicates were done (three wells in the plate). Significance was determined by one-way ANOVA using GraphPad Prism, n = 3. *p < 0.05, **p < 0.01, and ***p < 0.001.

### Mitochondrial respiration rate measurements

The respiration rate of the RPTECs were determined using the Agilent Seahorse XF-96 Extracellular Flux 374 Analyzer. The RPTECs were seeded in 96-well microplates at 35,000 cells per well and allowed to adhere overnight. The following day the cells were treated with either 30 µM TDF or in complete media for 2 days. One hour before plate reading, the media was switched to a media without bicarbonate or phenol red, 1 mM pyruvate, 2 mM glutamine, 10 mM glucose, 30 µM TDF and allowed to incubate at 37 °C without CO_2_. The drug injection ports were filled with oligomycin (Port A, final 1.5 µM), FCCP (Port B, final 2.0 uM), and rotenone/antimycin A (Port C, final 0.5 uM). Respiration was measured three times before the first drug injection and after each drug injection to allow for reading stabilization. The exception was that six measurements were taken after oligomycin injection. Each biological replicate (one 96-well plate) contained 6 technical replicates. Oxygen consumption rates were obtained through the Wave software (Agilent). In order to calculate the basal respiration, M13 (first measurement after rotenone/antimycin A injection) was subtracted from M3 (measurement immediately before oligomycin injection). Proton leak was measured by subtracting M13 from M9 (last measurement after oligomycin injection). ATP-linked respiration was calculated by subtracting the proton leak from basal respiration. Maximal respiration was calculated by subtracting M13 from M10 (first measurement after FCCP injection). The spare reverse capacity was calculated by subtracting the basal respiration from the maximal respiration. The coupling efficiency was calculated by diving the ATP-linked respiration by the basal respiration and multiplying by 100. The cell respiratory control ratio was calculated by dividing the maximal respiration by the proton leak. Lastly, the ATP-linked and maximal respiration ratio was calculated by diving the ATP-linked ratio by the maximal respiration. The values were averaged over separate experiments, n = 3. Significance was determined by one-way ANOVA using GraphPad Prism *p < 0.05, **p < 0.01, and ***p < 0.001.

## Supplementary information

Supplementary information.

## References

[1] Fischl MA (1987). The efficacy of azidothymidine (AZT) in the treatment of patients with AIDS and AIDS-related complex. A double-blind, placebo-controlled trial. N Engl J Med.

[2] Ray M (2010). The effect of combined antiretroviral therapy on the overall mortality of HIV-infected individuals. AIDS.

[3] Antiretroviral Therapy Cohort, C. (2008). Life expectancy of individuals on combination antiretroviral therapy in high-income countries: a collaborative analysis of 14 cohort studies. Lancet.

[4] Palella FJ (2006). Mortality in the highly active antiretroviral therapy era: changing causes of death and disease in the HIV outpatient study. J Acquir Immune Defic Syndr.

[5] Palella FJ (1998). Declining morbidity and mortality among patients with advanced human immunodeficiency virus infection. HIV Outpatient Study Investigators. N Engl J Med.

[6] Johnson AA (2001). Toxicity of antiviral nucleoside analogs and the human mitochondrial DNA polymerase. J Biol Chem.

[7] Bailey CM, Anderson KS (2010). A mechanistic view of human mitochondrial DNA polymerase gamma: providing insight into drug toxicity and mitochondrial disease. Biochim Biophys Acta.

[8] Lewis W, Day BJ, Copeland WC (2003). Mitochondrial toxicity of NRTI antiviral drugs: an integrated cellular perspective. Nat Rev Drug Discov.

[9] Brinkman K, ter Hofstede HJ, Burger DM, Smeitink JA, Koopmans PP (1998). Adverse effects of reverse transcriptase inhibitors: mitochondrial toxicity as common pathway. AIDS.

[10] Montaner JS (2004). Nucleoside-related mitochondrial toxicity among HIV-infected patients receiving antiretroviral therapy: insights from the evaluation of venous lactic acid and peripheral blood mitochondrial DNA. Clin Infect Dis.

[11] Moyle, G. Clinical manifestations and management of antiretroviral nucleoside analog-related mitochondrial toxicity. *Clin Ther* 22, 911–936; discussion 898, 10.1016/S0149-2918(00)80064-8 (2000). 10.1016/S0149-2918(00)80064-8 10972629

[12] Fernandez-Fernandez B (2011). Tenofovir nephrotoxicity: 2011 update. AIDS Res Treat.

[13] Kohler JJ (2009). Tenofovir renal toxicity targets mitochondria of renal proximal tubules. Lab Invest.

[14] Tourret J, Deray G, Isnard-Bagnis C (2013). Tenofovir effect on the kidneys of HIV-infected patients: a double-edged sword?. J Am Soc Nephrol.

[15] Birkus G (2002). Tenofovir diphosphate is a poor substrate and a weak inhibitor of rat DNA polymerases alpha, delta, and epsilon*. Antimicrob Agents Chemother.

[16] Lee H, Hanes J, Johnson KA (2003). Toxicity of nucleoside analogues used to treat AIDS and the selectivity of the mitochondrial DNA polymerase. Biochemistry.

[17] Chang CN, Skalski V, Zhou JH, Cheng YC (1992). Biochemical pharmacology of (+)- and (−)-2′,3′-dideoxy-3′-thiacytidine as anti-hepatitis B virus agents. J Biol Chem.

[18] Parker WB (1993). Metabolism of carbovir, a potent inhibitor of human immunodeficiency virus type 1, and its effects on cellular metabolism. Antimicrob Agents Chemother.

[19] Young MJ (2017). Off-Target Effects of Drugs that Disrupt Human Mitochondrial DNA Maintenance. Front Mol Biosci.

[20] Bianchi J (2013). PrimPol bypasses UV photoproducts during eukaryotic chromosomal DNA replication. Mol Cell.

[21] Garcia-Gomez S (2013). PrimPol, an archaic primase/polymerase operating in human cells. Mol Cell.

[22] Iyer LM, Koonin EV, Leipe DD, Aravind L (2005). Origin and evolution of the archaeo-eukaryotic primase superfamily and related palm-domain proteins: structural insights and new members. Nucleic Acids Res.

[23] Mouron S (2013). Repriming of DNA synthesis at stalled replication forks by human PrimPol. Nat Struct Mol Biol.

[24] Wan L (2013). hPrimpol1/CCDC111 is a human DNA primase-polymerase required for the maintenance of genome integrity. EMBO Rep.

[25] Kobayashi K (2016). Repriming by PrimPol is critical for DNA replication restart downstream of lesions and chain-terminating nucleosides. Cell Cycle.

[26] Schiavone D (2016). PrimPol Is Required for Replicative Tolerance of G Quadruplexes in Vertebrate Cells. Mol Cell.

[27] Svikovic, S. *et al*. R-loop formation during S phase is restricted by PrimPol-mediated repriming. *EMBO J* **38**, 10.15252/embj.201899793 (2019). 10.15252/embj.201899793 PMC6356060 30478192

[28] Mislak AC, Anderson KS (2016). Insights into the Molecular Mechanism of Polymerization and Nucleoside Reverse Transcriptase Inhibitor Incorporation by Human PrimPol. Antimicrob Agents Chemother.

[29] Li M (2016). The DNA Polymerase Gamma R953C Mutant Is Associated with Antiretroviral Therapy-Induced Mitochondrial Toxicity. Antimicrob Agents Chemother.

[30] Matsui T, Yamamoto T, Wyder S, Zdobnov EM, Kadowaki T (2009). Expression profiles of urbilaterian genes uniquely shared between honey bee and vertebrates. BMC Genomics.

[31] Torregrosa-Munumer R (2017). PrimPol is required for replication reinitiation after mtDNA damage. Proc Natl Acad Sci USA.

[32] Maffioli SI (2017). Antibacterial Nucleoside-Analog Inhibitor of Bacterial RNA Polymerase. Cell.

[33] Selvaraj S (2014). Antiretroviral therapy-induced mitochondrial toxicity: potential mechanisms beyond polymerase-gamma inhibition. Clin Pharmacol Ther.

[34] Rechkoblit O (2016). Structure and mechanism of human PrimPol, a DNA polymerase with primase activity. Sci Adv.

[35] Calvo PA (2019). The invariant glutamate of human PrimPol DxE motif is critical for its Mn(2+)-dependent distinctive activities. DNA Repair (Amst).

[36] Guilliam, T. A. & Doherty, A. J. PrimPol-Prime Time to Reprime. *Genes (Basel)* **8**, 10.3390/genes8010020 (2017). 10.3390/genes8010020 PMC5295015 28067825

[37] Pilzecker B (2016). PrimPol prevents APOBEC/AID family mediated DNA mutagenesis. Nucleic Acids Res.

[38] Keen BA, Jozwiakowski SK, Bailey LJ, Bianchi J, Doherty AJ (2014). Molecular dissection of the domain architecture and catalytic activities of human PrimPol. Nucleic Acids Res.

[39] Martinez-Jimenez MI, Calvo PA, Garcia-Gomez S, Guerra-Gonzalez S, Blanco L (2018). The Zn-finger domain of human PrimPol is required to stabilize the initiating nucleotide during DNA priming. Nucleic Acids Res.

[40] Graves SW, Johnson AA, Johnson KA (1998). Expression, purification, and initial kinetic characterization of the large subunit of the human mitochondrial DNA polymerase. Biochemistry.

[41] Kati WM, Johnson KA, Jerva LF, Anderson KS (1992). Mechanism and fidelity of HIV reverse transcriptase. J Biol Chem.

[42] Patel SS, Wong I, Johnson KA (1991). Pre-steady-state kinetic analysis of processive DNA replication including complete characterization of an exonuclease-deficient mutant. Biochemistry.

[43] Keen BA, Bailey LJ, Jozwiakowski SK, Doherty AJ (2014). Human PrimPol mutation associated with high myopia has a DNA replication defect. Nucleic Acids Res.

[44] Ryder SP, Recht MI, Williamson JR (2008). Quantitative analysis of protein-RNA interactions by gel mobility shift. Methods Mol Biol.

[45] Huynh K, Partch CL (2015). Analysis of protein stability and ligand interactions by thermal shift assay. Curr Protoc Protein Sci.

[46] Niesen FH, Berglund H, Vedadi M (2007). The use of differential scanning fluorimetry to detect ligand interactions that promote protein stability. Nat Protoc.

[47] Wieser M (2008). hTERT alone immortalizes epithelial cells of renal proximal tubules without changing their functional characteristics. Am J Physiol Renal Physiol.

[48] Zhao X (2017). Tenofovir and adefovir down-regulate mitochondrial chaperone TRAP1 and succinate dehydrogenase subunit B to metabolically reprogram glucose metabolism and induce nephrotoxicity. Sci Rep.

[49] Zhang J (2012). Measuring energy metabolism in cultured cells, including human pluripotent stem cells and differentiated cells. Nat Protoc.

[50] Kohler JJ, Lewis W (2007). A brief overview of mechanisms of mitochondrial toxicity from NRTIs. Environ Mol Mutagen.

[51] White AJ (2001). Mitochondrial toxicity and HIV therapy. Sex Transm Infect.

[52] Quinet, A. *et al*. PRIMPOL-Mediated Adaptive Response Suppresses Replication Fork Reversal in BRCA-Deficient Cells. *Mol Cell*, 10.1016/j.molcel.2019.10.008 (2019). 10.1016/j.molcel.2019.10.008 PMC7007862 31676232

[53] Guilliam TA (2017). Molecular basis for PrimPol recruitment to replication forks by RPA. Nat Commun.

[54] Martinez-Jimenez MI, Lahera A, Blanco L (2017). Human PrimPol activity is enhanced by RPA. Sci Rep.

[55] Fang JL, Beland FA (2009). Long-term exposure to zidovudine delays cell cycle progression, induces apoptosis, and decreases telomerase activity in human hepatocytes. Toxicol Sci.

[56] Krasich R, Copeland WC (2017). DNA polymerases in the mitochondria: A critical review of the evidence. Front Biosci (Landmark Ed).

[57] Zafar MK, Ketkar A, Lodeiro MF, Cameron CE, Eoff RL (2014). Kinetic analysis of human PrimPol DNA polymerase activity reveals a generally error-prone enzyme capable of accurately bypassing 7,8-dihydro-8-oxo-2′-deoxyguanosine. Biochemistry.

[58] Xu W, Zhao W, Morehouse N, Tree MO, Zhao L (2019). Divalent Cations Alter the Rate-Limiting Step of PrimPol-Catalyzed DNA Elongation. J Mol Biol.

[59] Hoehener C, Hug I, Nowacki M (2018). Dicer-like Enzymes with Sequence Cleavage Preferences. Cell.

[60] Salter JD, Smith HC (2018). Modeling the Embrace of a Mutator: APOBEC Selection of Nucleic Acid Ligands. Trends Biochem Sci.

[61] Tian L, Kim MS, Li H, Wang J, Yang W (2018). Structure of HIV-1 reverse transcriptase cleaving RNA in an RNA/DNA hybrid. Proc Natl Acad Sci USA.

[62] Chuprina VP (1991). Sequence effects on local DNA topology. Proc Natl Acad Sci USA.

[63] De Clercq E (2003). Clinical potential of the acyclic nucleoside phosphonates cidofovir, adefovir, and tenofovir in treatment of DNA virus and retrovirus infections. Clin Microbiol Rev.

[64] Anderson AC (2003). The process of structure-based drug design. Chem Biol.

[65] Blanco L (2019). Mechanism of DNA primer synthesis by human PrimPol. Enzymes.

[66] Diaz-Talavera A (2019). A cancer-associated point mutation disables the steric gate of human PrimPol. Sci Rep.

[67] Rudd SG, Bianchi J, Doherty AJ (2014). PrimPol-A new polymerase on the block. Mol Cell Oncol.

[68] Tuttle DL (2002). Increased replication of non-syncytium-inducing HIV type 1 isolates in monocyte-derived macrophages is linked to advanced disease in infected children. AIDS Res Hum Retroviruses.

[69] Tropea JE, Cherry S, Waugh DS (2009). Expression and purification of soluble His(6)-tagged TEV protease. Methods Mol Biol.

[70] Rooney JP (2015). PCR based determination of mitochondrial DNA copy number in multiple species. Methods Mol Biol.

