# Supplementary material for: Identifying the role of PrimPol in TDF-induced toxicity and implications of its loss of function mutation in an HIV+ patient
Source: Sci Rep. 2020 Jun 9;10:9343. doi: 10.1038/s41598-020-66153-z (PMC7283272; doi:10.1038/s41598-020-66153-z)
Supplement: Supplementary file 1 — Supplementary information. [file 41598_2020_66153_MOESM1_ESM.pdf]

Supplemental Figures, Tables, Legends

Identifying the role of PrimPol in TDF-induced toxicity and implications of its loss of function mutation in an HIV+ patient

Vincent N. Duong,[1] Lei Zhou,[2] María I. Martínez-Jiménez,[3] Linh He,[1] Moises Cosme,[2] Luis Blanco,[3,*] Elijah Paintsil,[1,2,4,*] Karen S. Anderson[1,5,*]

[1]Department of Pharmacology, Yale School of Medicine, 06510 New Haven, Connecticut, USA
[2]Department of Pediatrics, Yale School of Medicine, 06510 New Haven, Connecticut, USA
[3]Centro de Biología Molecular Severo Ochoa, CSIC-UAM, 28049 Madrid, Spain
[4]Department of Epidemiology & Public Health, Yale School of Medicine, 06510 New Haven, Connecticut, USA
[5]Department of Molecular Biophysics and Biochemistry, Yale University, 06510 New Haven, Connecticut, United States of America
[*]Correspondence: karen.anderson@yale.edu, elijah.paintsil@yale.edu, lblanco@cbm.csic.es

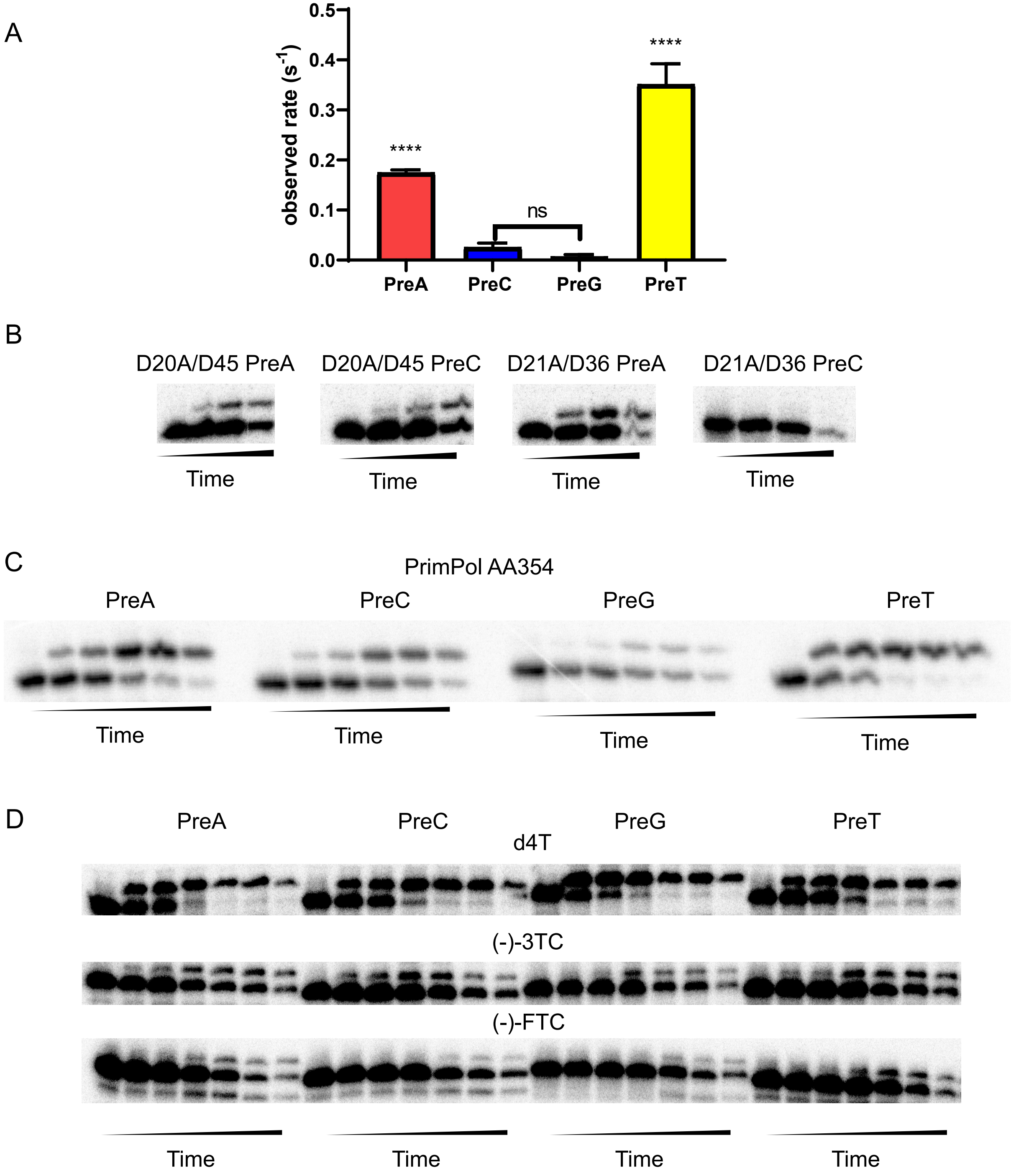
A
0.5
0.4
0.3
0.2
0.1
0.0
observed rate (s-1)
****
****
ns
PreA
PreC
PreG
PreT
B
D20A/D45 PreA
D20A/D45 PreC
D21A/D36 PreA
D21A/D36 PreC
Time
Time
Time
Time
C
PrimPol AA354
PreA
PreC
PreG
PreT
Time
Time
Time
Time
D
PreA
PreC
PreG
PreT
d4T
(-)-3TC
(-)-FTC
Time
Time
Time
Time

Fig. S1

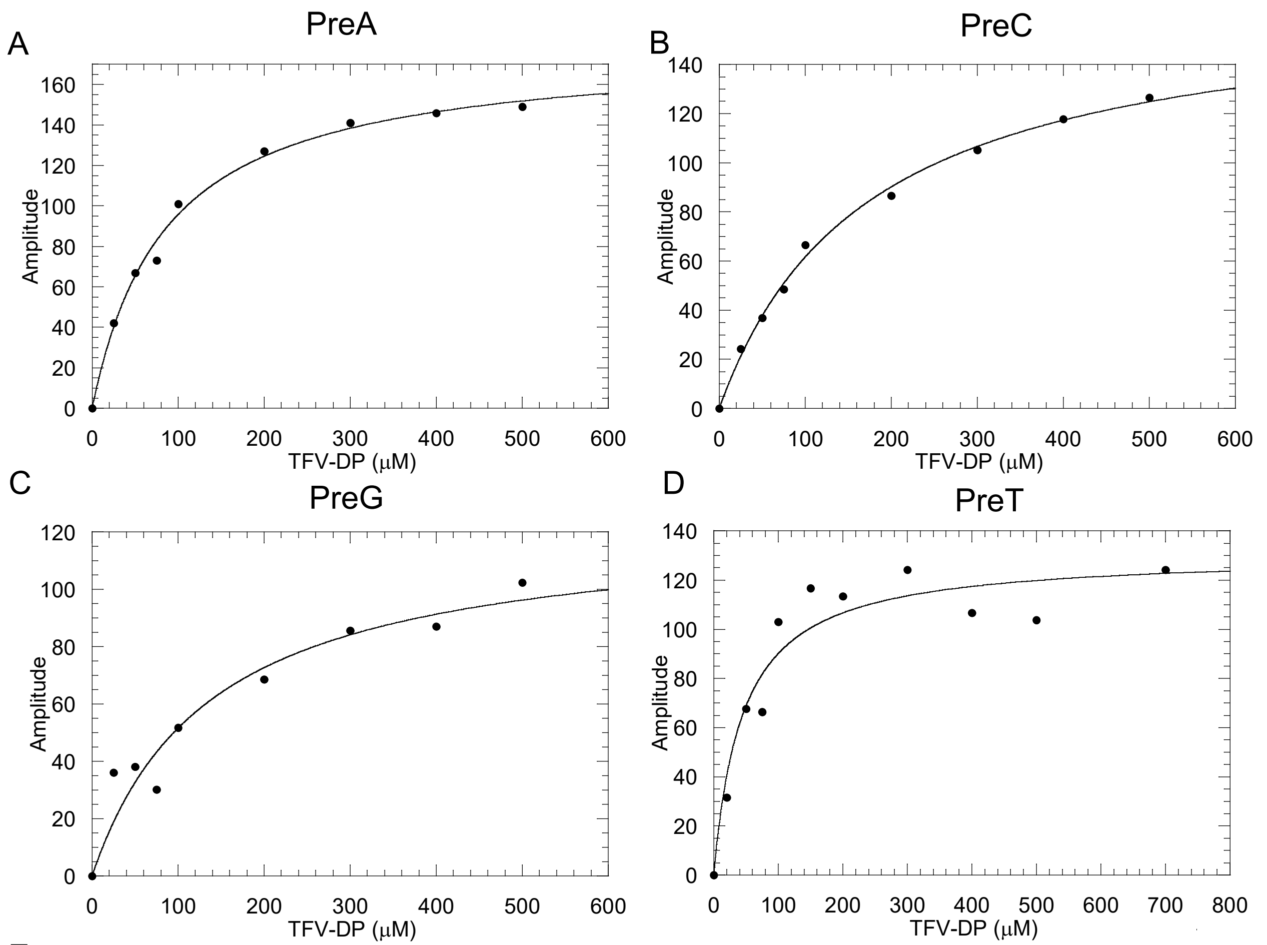

E

TFV-DP (μM)	rate (s^{-1})			
	PreA	PreC	PreG	PreT
25	1.9 x 10^{-3}	1.2 x 10^{-3}	1.8 x 10^{-3}	6.1 x 10^{-3}
50	1.1 x 10^{-3}	1.8 x 10^{-3}	2.2 x 10^{-3}	2.1 x 10^{-3}
75	2.1 x 10^{-3}	1.2 x 10^{-3}	1.6 x 10^{-3}	2.2 x 10^{-3}
100	1.5 x 10^{-3}	1.1 x 10^{-3}	1.5 x 10^{-3}	3.0 x 10^{-3}
150	N/A	N/A	N/A	2.7 x 10^{-3}
200	1.4 x 10^{-3}	1.3 x 10^{-3}	1.1 x 10^{-3}	2.9 x 10^{-3}
300	1.4 x 10^{-3}	1.3 x 10^{-3}	1.0 x 10^{-3}	4.6 x 10^{-3}
400	1.4 x 10^{-3}	1.3 x 10^{-3}	1.1 x 10^{-3}	4.5 x 10^{-3}
500	1.8 x 10^{-3}	1.3 x 10^{-3}	0.9 x 10^{-3}	4.9 x 10^{-3}
700	N/A	N/A	N/A	4.0 x 10^{-3}
1000	N/A	N/A	N/A	3.8 x 10^{-3}

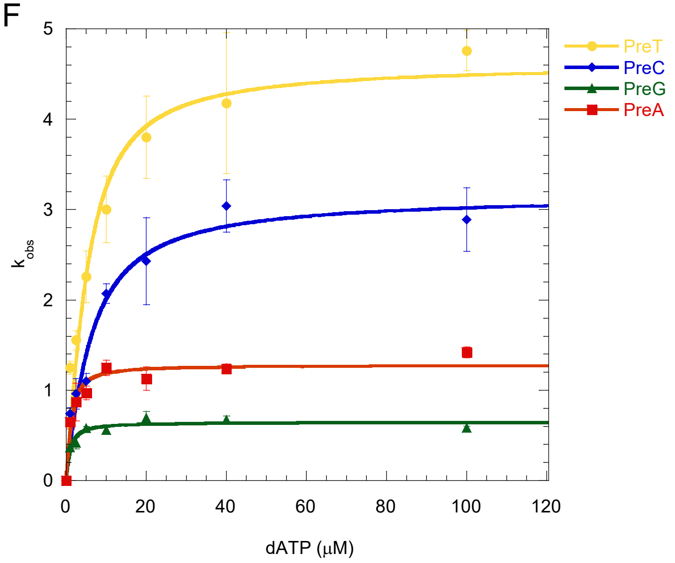

Fig. S2

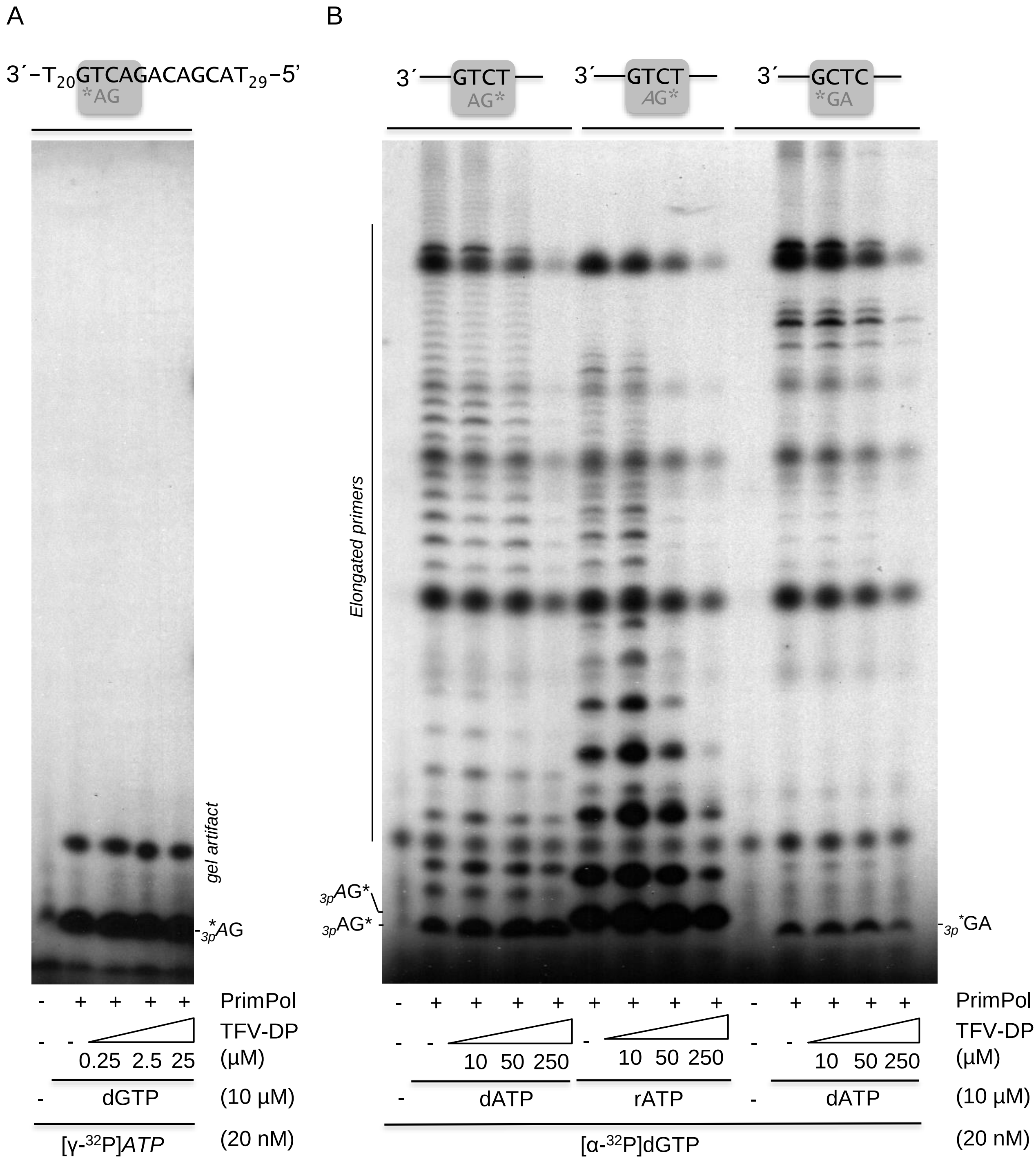
A
B
3´-T20GTCAGACAGCAT29-5’
*AG
3´—GTCT—
AG*
3´—GTCT—
AG*
3´—GCTC—
*GA
Elongated primers
gel artifact
3pAG*
3pAG*
3p*AG
3p*GA
PrimPol
TFV-DP (µM)
0.25 2.5 25
10 50 250
dGTP
dATP
rATP
(10 µM)
[γ-32P]ATP
[α-32P]dGTP
(20 nM)

Fig. S3

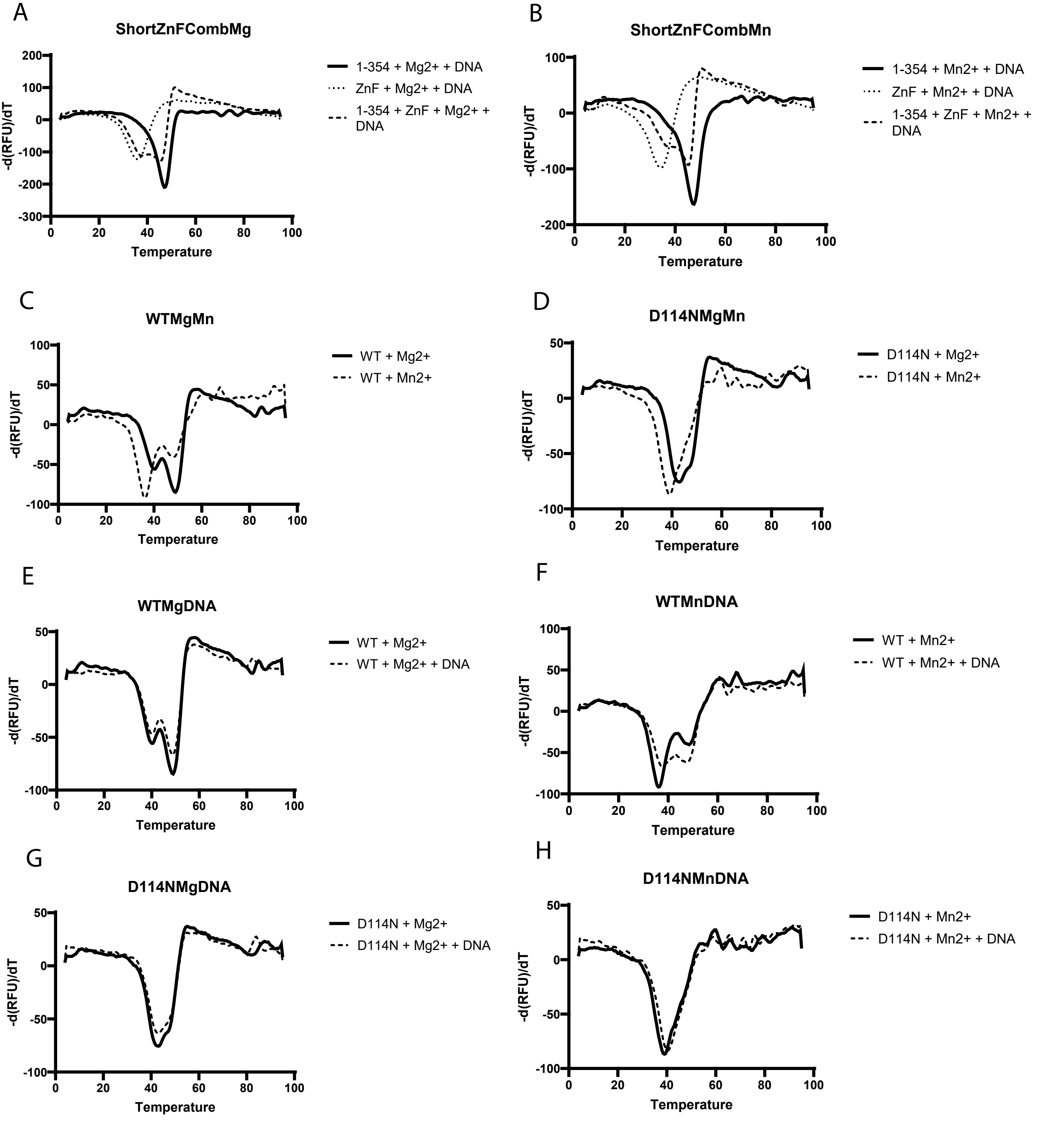
A
ShortZnFCombMg
1-354 + Mg2+ + DNA
ZnF + Mg2+ + DNA
1-354 + ZnF + Mg2+ + DNA
-d(RFU)/dT
Temperature
B
ShortZnFCombMn
1-354 + Mn2+ + DNA
ZnF + Mn2+ + DNA
1-354 + ZnF + Mn2+ + DNA
-d(RFU)/dT
Temperature
C
WTMgMn
WT + Mg2+
WT + Mn2+
-d(RFU)/dT
Temperature
D
D114NMgMn
D114N + Mg2+
D114N + Mn2+
-d(RFU)/dT
Temperature
E
WTMgDNA
WT + Mg2+
WT + Mg2+ + DNA
-d(RFU)/dT
Temperature
F
WTMnDNA
WT + Mn2+
WT + Mn2+ + DNA
-d(RFU)/dT
Temperature
G
D114NMgDNA
D114N + Mg2+
D114N + Mg2+ + DNA
-d(RFU)/dT
Temperature
H
D114NMnDNA
D114N + Mn2+
D114N + Mn2+ + DNA
-d(RFU)/dT
Temperature

Fig. S4

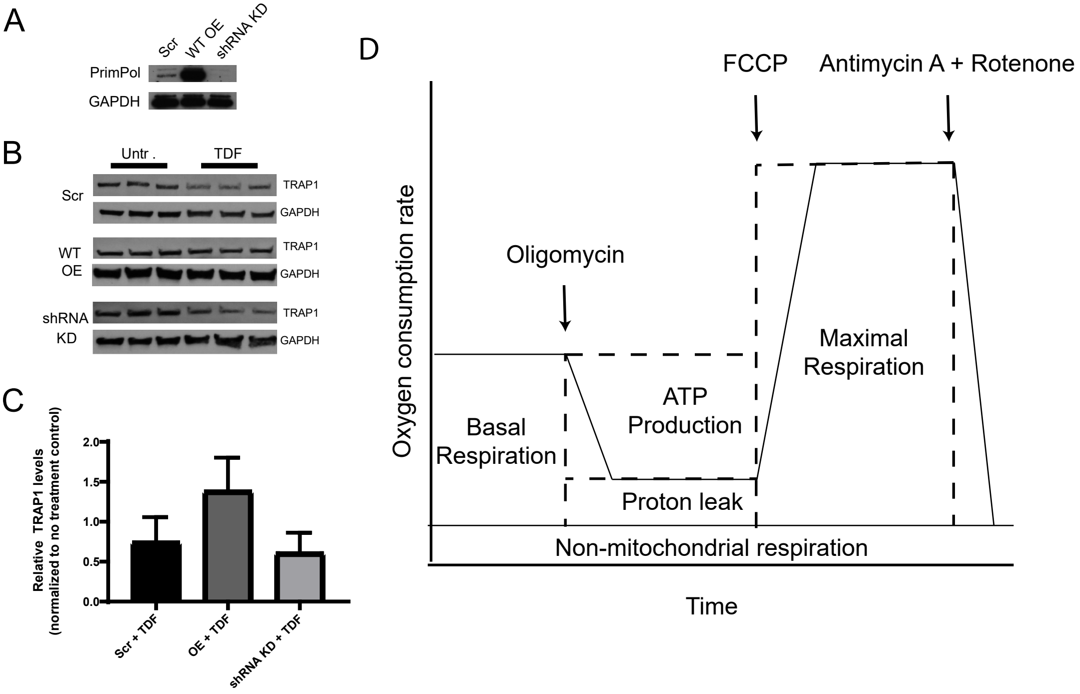
A
Scr
WT OE
shRNA KD
PrimPol
GAPDH
B
Untr .
TDF
Scr
TRAP1
GAPDH
WT
OE
TRAP1
GAPDH
shRNA
KD
TRAP1
GAPDH
C
Relative TRAP1 levels
(normalized to no treatment control)
2.0
1.5
1.0
0.5
0.0
Scr + TDF
OE + TDF
shRNA KD + TDF
D
FCCP
Antimycin A + Rotenone
Oxygen consumption rate
Oligomycin
Maximal
Respiration
ATP
Production
Basal
Respiration
Proton leak
Non-mitochondrial respiration
Time

Fig. S5

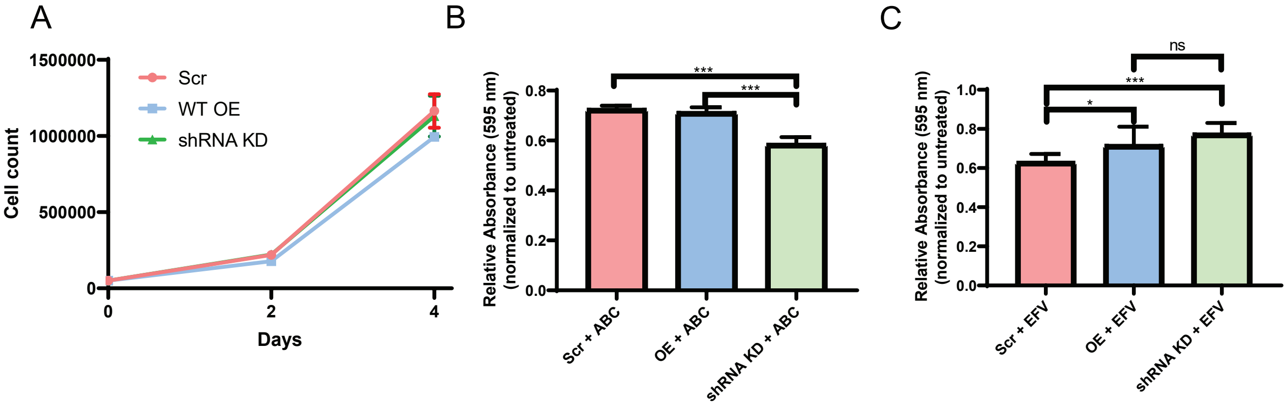

A
Cell count
1500000
1000000
500000
0
0
2
4
Days
Scr
WT OE
shRNA KD
B
Relative Absorbance (595 nm)
(normalized to untreated)
0.8
0.6
0.4
0.2
0.0
***
***
Scr + ABC
OE + ABC
shRNA KD + ABC
C
1.0
0.8
0.6
0.4
0.2
0.0
ns
***
*
Scr + EFV
OE + EFV
shRNA KD + EFV

Fig. S6

A

mtDNA

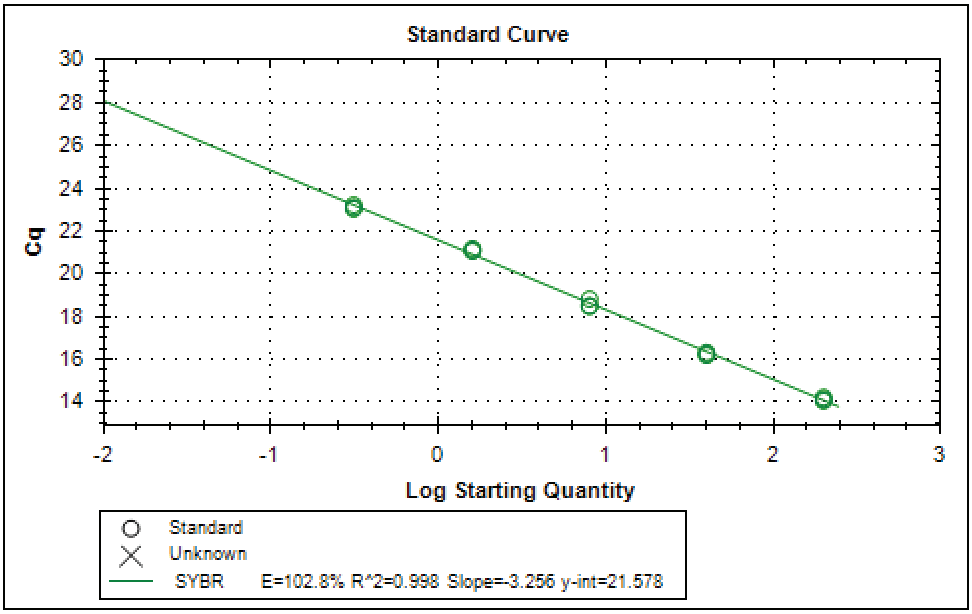

B

nucDNA

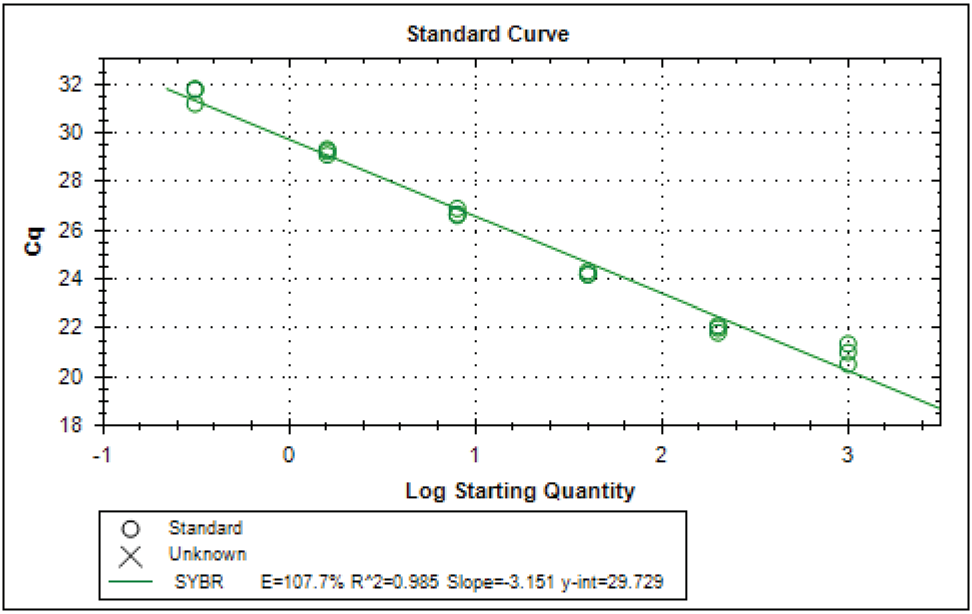

Fig. S7

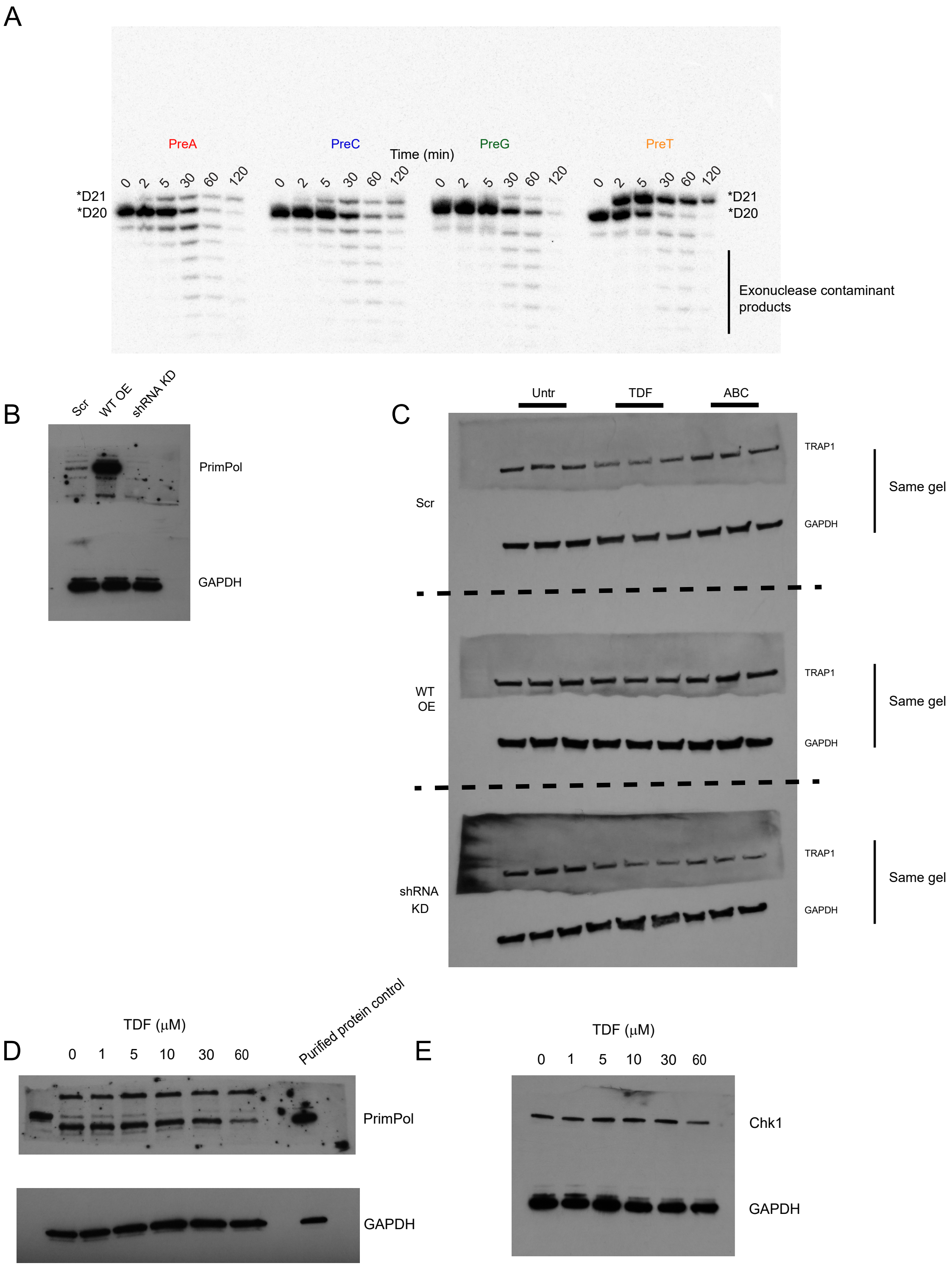
A
PreA
PreC
PreG
PreT
Time (min)
0 2 5 30 60 120
*D21
*D20
Exonuclease contaminant products
B
Scr
WT OE
shRNA KD
PrimPol
GAPDH
C
Untr
TDF
ABC
Scr
WT OE
shRNA KD
TRAP1
GAPDH
Same gel
D
TDF (μM)
0 1 5 10 30 60
Purified protein control
PrimPol
GAPDH
E
TDF (μM)
0 1 5 10 30 60
Chk1
GAPDH

Fig. S8

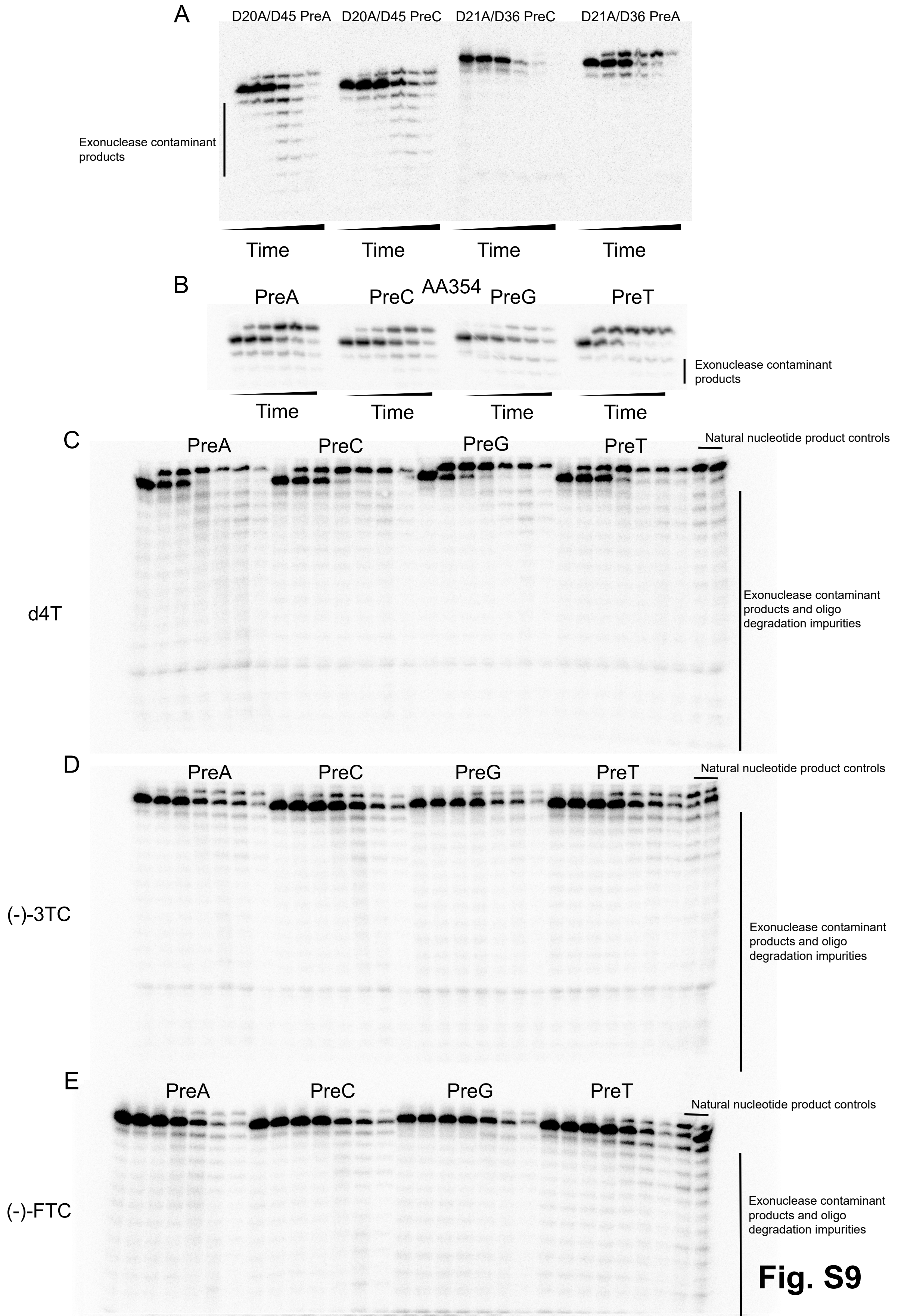
A
D20A/D45 PreA
D20A/D45 PreC
D21A/D36 PreC
D21A/D36 PreA
Exonuclease contaminant products
Time
Time
Time
Time
B
AA354
PreA
PreC
PreG
PreT
Exonuclease contaminant products
Time
Time
Time
Time
C
PreA
PreC
PreG
PreT
Natural nucleotide product controls
d4T
Exonuclease contaminant products and oligo degradation impurities
D
PreA
PreC
PreG
PreT
Natural nucleotide product controls
(-)-3TC
Exonuclease contaminant products and oligo degradation impurities
E
PreA
PreC
PreG
PreT
Natural nucleotide product controls
(-)-FTC
Exonuclease contaminant products and oligo degradation impurities

Fig. S9

NRTI	k_{pol} (s^{-1})	K_d (μM)	Inc. Eff. k_{pol}/K_d (s^{-1} μM^{-1})
TFV-DP	0.0037 ± 0.001	45.0 ± 13.7	8.2×10^{-5}
ddATP[a]	0.0138 ± 0.0008	15 ± 3	9.0×10^{-4}
AZT-TP[a]	0.0040 ± 0.0008	38 ± 6	1.0×10^{-4}
ddCTP[a]	0.033 ± 0.002	18 ± 4	2.0×10^{-3}
CBV-TP[a]	0.013 ± 0.002	~1	1.3×10^{-2}

Table S1.

Exp. 1A	WT + Mg^{2+}	WT + Mn^{2+}	D114N + Mg^{2+}	D114N + Mn^{2+}	WT + Mg^{2+} + DNA	WT + Mn^{2+} + DNA	D114N + Mg^{2+} + DNA	D114N + Mn^{2+} + DNA		
Min 1	40.08 ± 0.20	36.08 ± 0.20	42.83 ± 0.26	38.67 ± 0.26	40.00 ± 0.00	37.83 ± 0.41	42.58 ± 0.20	40.17 ± 0.26		
Min 2	48.83 ± 0.26	47.83 ± 0.41			48.92 ± 0.20	47.17 ± 0.26				
Exp. 1B	WT + Mg^{2+}	WT + Mn^{2+}	D114N + Mg^{2+}	D114N + Mn^{2+}	WT + Mg^{2+} + DNA	WT + Mn^{2+} + DNA	D114N + Mg^{2+} + DNA	D114N + Mn^{2+} + DNA		
Min 1	39.92 ± 0.20	36.25 ± 0.27	42.75 ± 0.27	38.75 ± 0.27	40.42 ± 0.20	38.00 ± 0.55	42.67 ± 0.26	40.00 ± 0.00		
Min 2	48.83 ± 0.26	47.83 ± 0.41			48.92 ± 0.20	47.17 ± 0.26				
Exp. 2	1-354 + Mg^{2+}	1-354 + Mn^{2+}	ZnF + Mg^{2+}	ZnF + Mn^{2+}	1-354 + Mg^{2+} + DNA	1-354 + Mn^{2+} + DNA	ZnF + Mg^{2+} + DNA	ZnF + Mn^{2+} + DNA	1-354 + ZnF + Mg^{2+} + DNA	1-354 + ZnF + Mn^{2+} + DNA
Min 1	47.33 ±0.29	47.33 ± 0.29	35.50 ± 0.00	33.50 ± 0.00	47.17 ± 0.29	47.33 ± 0.29	36.00 ± 0.00	34.00 ± 0.00	38.67 ± 0.58	38.50 ± 0.71
Min 2									35.15 ± 0.00	34.94 ± 0.29

Table S2.

Biological Replicate 1									
	mtDNA	nucDNA	Delta Ct	Relative mtDNA content [2*2^(DeltaCt)]				AVG mtDNA content	Normalized
Scr NTC TechRep 1	20.03	28.00	7.97	500.9496			SCR NTC	472.8759918	1
Scr TFV TechRep 1	20.18	28.19	8.00	513.314			SCR TDF	546.3161702	1.155305
Scr NTC TechRep 2	19.87	27.83	7.96	498.1751					
Scr TFV TechRep 2	20.04	28.04	8.00	510.6142					
Scr NTC TechRep 3	19.85	27.56	7.71	419.5033					
Scr TFV TechRep 3	19.72	27.99	8.26	615.0203					
WT NTC TechRep 1	19.68	27.55	7.87	468.6697			WT NTC	494.9494334	1
WT TFV TechRep 1	20.20	28.18	7.98	504.8834			WT TDF	467.8578512	0.945264
WT NTC TechRep 2	19.52	27.36	7.84	458.1809					
WT TFV TechRep 2	20.22	27.88	7.66	404.5132					
WT NTC TechRep 3	19.25	27.37	8.12	557.9977					
WT TFV TechRep 3	19.82	27.77	7.95	494.1769					
shRNA KD NTC TechRep 1	19.63	27.66	8.03	521.9029			SH1 NTC	549.407456	1
shRNA KD TFV TechRep 1	20.56	28.15	7.58	383.2727			SH1 TDF	410.1631384	0.746555
shRNA KD NTC TechRep 2	19.57	27.79	8.22	594.8089					
shRNA KD TFV TechRep 2	20.41	28.07	7.66	404.1841					
shRNA KD NTC TechRep 3	19.44	27.50	8.05	531.5106					
shRNA KD TFV TechRep 3	20.21	28.00	7.79	443.0327					

Biological Replicate 2									
	mtDNA	nucDNA	DeltaCt	Relative mtDNA content [2*2^(DeltaCt)]				AVG mtDNA content	Normalized
Scr NTC TechRep 1	19.26	26.93	7.67	408.2564			SCR NTC	364.6530655	1
Scr TFV TechRep 1	20.36	28.68	8.32	637.5225			SCR TDF	565.6129122	1.551099
Scr NTC TechRep 2	19.20	26.66	7.46	351.5148					
Scr TFV TechRep 2	20.27	28.28	8.01	514.7933					
Scr NTC TechRep 3	19.04	26.42	7.38	334.188					
Scr TFV TechRep 3	20.08	28.17	8.09	544.5229					
WT NTC TechRep 1	19.92	27.70	7.78	439.3915			WT NTC	448.4038351	1
WT TFV TechRep 1	19.89	28.02	8.13	559.7335			WT TDF	638.6335773	1.424238
WT NTC TechRep 2	19.98	27.63	7.65	400.3327					
WT TFV TechRep 2	19.66	27.99	8.33	642.3445					
WT NTC TechRep 3	19.73	27.71	7.98	505.4873					
WT TFV TechRep 3	19.32	27.80	8.48	713.8227					
shRNA KD NTC TechRep 1	18.98	27.00	8.03	521.7694			SH1 NTC	531.8546058	1
shRNA KD TFV TechRep 1	19.98	27.85	7.87	467.8371			SH1 TDF	483.4297483	0.908951
shRNA KD NTC TechRep 2	18.84	26.81	7.97	501.5229					
shRNA KD TFV TechRep 2	19.96	27.81	7.86	464.113					
shRNA KD NTC TechRep 3	18.61	26.77	8.16	572.2715					
shRNA KD TFV TechRep 3	19.63	27.64	8.02	518.3391					

Biological Replicate 3									
	mtDNA	nucDNA	DeltaCt	Relative mtDNA content [2*2^(DeltaCt)]				AVG mtDNA content	Normalized
Scr NTC TechRep 1	19.47	28.16	8.69	827.3045			SCR NTC	857.0247043	1
Scr TFV TechRep 1	19.52	28.62	9.10	1097.552			SCR TDF	1307.58689	1.525728
Scr NTC TechRep 2	19.42	28.25	8.83	911.942					
Scr TFV TechRep 2	19.19	28.68	9.49	1440.672					
Scr NTC TechRep 3	19.41	28.11	8.70	831.8276					
Scr TFV TechRep 3	19.28	28.72	9.44	1384.537					
WT NTC TechRep 1	19.79	28.36	8.57	757.6055			WT NTC	911.2695513	1
WT TFV TechRep 1	19.37	28.36	8.99	1018.564			WT TDF	1189.202425	1.304995
WT NTC TechRep 2	19.58	28.39	8.81	899.1471					
WT TFV TechRep 2	19.24	28.61	9.37	1319.842					
WT NTC TechRep 3	19.40	28.47	9.07	1077.056					
WT TFV TechRep 3	19.19	28.45	9.26	1229.201					
shRNA KD NTC TechRep 1	19.45	28.28	8.83	909.1081			SH1 NTC	978.6155981	1
shRNA KD TFV TechRep 1	19.28	27.82	8.53	741.8484			SH1 TDF	794.2554558	0.811611
shRNA KD NTC TechRep 2	19.25	28.34	9.09	1089.003					
shRNA KD TFV TechRep 2	19.22	27.83	8.61	782.605					
shRNA KD NTC TechRep 3	19.21	28.08	8.87	937.7354					
shRNA KD TFV TechRep 3	19.09	27.84	8.75	858.313					

Table S3.

Figure S1. The preceding nucleotide preference is replicable in other sequences, occurs without the zinc finger, and is unique to TFV-DP (see Fig. 1).

A) Statistical significance of the estimated rates for TFV-DP incorporation in Fig. 1C and 1D using the one-way ANOVA test, **** = $p<0.0001$. B) Denaturing PAGE showing the preceding nucleotide preference for TFV-DP incorporation by PrimPol is present for two independent primer/template sequences, D20A/D45 (left two gels) and D21/D36 (right two gels). In both cases, PreA TFV-DP incorporation is favored over PreC. C) The preceding nucleotide preference also occurs with the isolated polymerase domain of PrimPol (amino acids 1-354) using the D20A/D45 primer/template. D) The preceding nucleotide preference was tested using different NRTIs, d4T (dT analog, D22T/D45, top), (-)-3TC (dC analog, D23C/D45, middle), and (-)-FTC (dC analog, D23C/D45, bottom). In all cases there was not a strong preceding nucleotide preference, demonstrating that the effect observed with TFV-DP is unique in the NRTIs tested.

Figure S2. Determining the kinetic parameters of TFV-DP and dATP incorporation by WT PrimPol dependent on the preceding nucleotide in the primer strand (see Table 1).

In the case of TFV-DP incorporation by PrimPol, the amplitude of product formation varied with TFV-DP concentration. A-D) The kinetic parameters of TFV-DP incorporation by PrimPol were calculated by plotting the amplitude of single turnover experiments with PrimPol and TFV-DP against the concentrations of TFV-DP used. The K_d was determined by the concentration at half of the amplitude max. E) The similar rates across TFV-DP concentrations were averaged to determine the k_{pol}. F) The k_{pol} and K_d of dATP incorporation by PrimPol was determined by plotting the rates of each single turnover experiment against the concentration of TFV-DP used in each experiment.

Table S1. Comparison of incorporation efficiencies of NRTIs by PrimPol (see Table 1).

Values for TFV-DdP incorporation by PrimPol under optimal conditions (preceding dT in the primer) are compared to the incorporation efficiency values of other NRTIs by PrimPol. The errors represent the standard error values of the parameters that corresponds to a confidence level of 68.3%, or to one standard deviation. [a]Values obtained from Ref. 28.

Figure S3. TFV-DP is able to reduce the product length of primers during elongation but unable to inhibit dimer formation (see Fig. 1).

A) Dimer formation during primer initiation by PrimPol was monitored in various concentrations of TFV-DP. TFV-DP did not compete with ATP even at excess

concentrations. B) The interference of TFV-DP in primer elongation by PrimPol was monitored. Using a 3'-GTCT-5' and a 3'-GCTC-5' substrate, TFV-DP was added at different concentrations in competition with dATP or ATP.

Figure S4. Thermal shift assay of WT and D114N PrimPol dependent on the presence of Mg^{2+} or Mn^{2+} and DNA (see Fig. 4G).

WT or D114N PrimPol (5 µM) was mixed with either $MgCl_2$ or $MnCl_2$ (10 µM) and dsDNA (5 µM) and the unfolding of the protein was monitored through fluorescence by SYPRO orange (A-H). A) Comparison of the polymerase domain (1-354) alone, zinc finger domain alone, and a stoichiometric mixture of both complexes in the presence of DNA with either Mg^{2+} or B) Mn^{2+}. C) Comparison of the melting temperatures with Mg^{2+} to Mn^{2+} present without DNA of WT or D) D114N PrimPol. E) Observing the effects of the presence of DNA on the melting temperature of WT PrimPol with either Mg^{2+} or F) Mn^{2+}. G) Observing the effects of the presence of DNA on the melting temperature of D114N PrimPol with either Mg^{2+} or H) Mn^{2+}.

Table S2. Melting temperatures of WT and D114N PrimPol dependent on the presence of Mg^{2+} or Mn^{2+} and DNA (see Fig. S3, 4G).

WT or D114N PrimPol (5 µM) was mixed with either $MgCl_2$ or $MnCl_2$ (10 µM) and dsDNA (5 µM) and the unfolding of the protein was monitored through fluorescence by SYPRO orange. The derivatives of the relative fluorescence units were plotted against temperature, the local minima identified, and the corresponding temperatures were recorded. Numbers are shown as the mean ± SD. For experiment 1A and 1B, n=6, and for experiment 2, n=3.

Figure S5. TRAP1 as an indicator of affected metabolism in PrimPol knockdown RPTECs (see Fig. 5).

A) Western blotting of PrimPol of the scrambled, wild-type PrimPol overexpression, and shRNA knockdown renal proximal tubule epithelial cell lines. B) Immunoblotting of TRAP1 in RPTECs after treatment of 30 µM TDF for 3 days. The lanes for each treatment represent a technical replicate, n=3. C) Quantified relative amounts of TRAP1 levels across 3 biological replicates. The normalized levels of TRAP1 to GAPDH were compared for each cell strain to the untreated control. The shRNA and scrambled cell lines show a downward, but statistically nonsignificant (one-way ANOVA), trend of TRAP1 levels compared to the PrimPol overexpression strain. D) Seahorse XF Analyzer MitoStress assay schematic to measure parameters related to mitochondrial respiration.

Figure S6. Modulating PrimPol does not affect cell viability and validating the effects of PrimPol levels on NRTI-caused toxicity (see Fig. 5).

A) Cells were seeded in 6-well plate at a low density and then counted after 2 and 4 days, n=3. B) The PrimPol RPTEC strains were treated with abacavir over 5 days and cellular proliferation was monitored using the MTT assay, n=3. C) As a control, the cells were treated with efavirenz, an NNRTI, over 5 days and the MTT assay was used to assess the toxicity, n=3. Significance was determined by one-way ANOVA, * = $p<0.05$, ** = $p<0.01$, and *** = $p<0.001$.

Figure S7. Standard curves for qPCR quantification of mtDNA.

The target DNA, A) mtDNA or B) nucDNA, was serial diluted and the cycle threshold was plotted against the concentration of DNA.

Table S3. Raw data for relative mtDNA quantification

Relative mtDNA content of RPTECs treated with TDF was calculated through qPCR. C_T values were obtained and relative mtDNA was calculated by subtracting the C_T of mtDNA from nucDNA. The final value was then determined by the equation $mtDNA = 2 * 2^{\Delta C_T}$. Those values were then normalized to the no treatment control. For each biological replicate, three technical replicates were conducted.

Figure S8. Full gels and blots for this study.

For Western blots, polyacrylamide gels were cut before membrane transfer. A) Fig. 1C. B) Fig. S5A, Samples were run on the same gel and corresponding molecular weights were cut out before transfer. C) Fig. S5B Samples were run on the same gel and corresponding molecular weights were cut out before transfer, as denoted by the solid line. Different gels are separated by the dashed line. All membranes were exposed at the same time. D, E) Fig. 5I, Samples were run on the same gel and corresponding molecular weights were cut out before transfer.

Figure S9. Full gels for Figure S1.

A) Full gel for Fig. S1B. B) Full gel for Fig. S1C. C-E) Full gels for Fig. S1D.
